# Synergistic Antitumor Immunotherapy via Mitochondria Regulation in Macrophages and Tumor Cells by an Iridium Photosensitizer

**DOI:** 10.1021/acscentsci.4c02156

**Published:** 2025-03-11

**Authors:** Shumeng Li, Hao Yuan, Xiu-Zhi Yang, Xinyu Xu, Wenhao Yu, Yanping Wu, Shankun Yao, Jin Xie, Weijiang He, Zijian Guo, Yuncong Chen

**Affiliations:** † State Key Laboratory of Coordination Chemistry, School of Chemistry and Chemical Engineering, Chemistry and Biomedicine Innovation Center (ChemBIC), ChemBioMed Interdisciplinary Research Center, Nanjing University, Nanjing 210023, Jiangsu, P.R. China; ‡ Department of Cardiothoracic Surgery, Nanjing Drum Tower Hospital, Medical School, Nanjing University, Nanjing 210008, Jiangsu, P.R. China

## Abstract

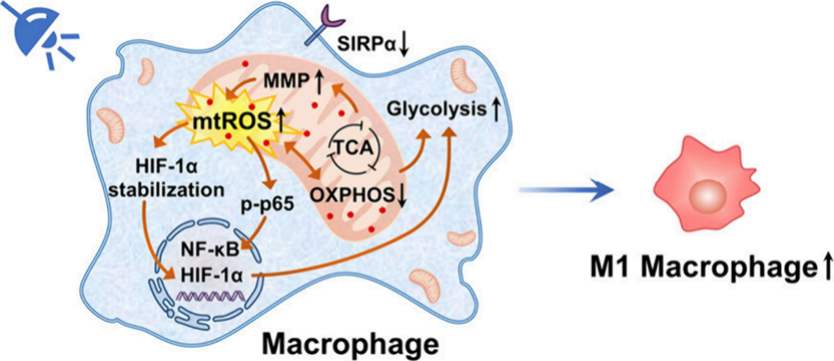

Mitochondrial targeting has emerged as an attractive method for antitumor treatment. However, most of the mitochondria targeted drugs focused on inhibiting tumor cells, while their potential for activation of immune responses in the tumor microenvironment has rarely been described. In this study, we report a photosensitive iridium complex **MitoIrL2**, which enabled the simultaneous mitochondrial modulation of macrophages and tumor cells to achieve synergistic antitumor immunity. The adjustment of the mitochondrial respiratory chain, HIF-1α, and the NF-κB pathway in macrophages drove the metabolic reprogramming from oxidative phosphorylation (OXPHOS) to glycolysis, converting protumor M2 into the antitumor M1 phenotype. Downregulated expression of immunosuppressive checkpoint SIRPα has also been observed on macrophages. Meanwhile, the mitochondrial targeting **MitoIrL2** enhanced the immunogenic cell death of tumor cells and reversed the immunosuppressive tumor microenvironment, which activated the systemic immune response and established long-term immune memory *in vivo*. This work illustrates a promising strategy to simultaneously regulate macrophages toward the antitumor phenotype and enhance immunogenic cell death in tumor cells for synergistic antitumor immunotherapy.

## Abstract 2

A mitochondrial targeting iridium photosensitizer could achieve direct immune activation of both macrophages and tumor cells, synergistically enhancing antitumor immunity via mitochondrial modulation.

## Introduction

Mitochondria, as critical subcellular compartments for many important metabolic and signaling processes,^1,2^ have received great attention in recent decades.^3,4^ Based on their core roles as the powerhouse of cells,^5^ a large number of mitochondria-targeting antitumor drugs have been developed.^6−8^ Mitochondria are also closely related to the immune response in the tumor microenvironment. Notably, mitochondria are necessary for immune cells to establish and sustain their phenotype and function,^9,10^ while various mitochondrial constituents and metabolites can act as damage-associated molecular patterns (DAMPs) and promote inflammation.^11,12^ However, most of the current studies only focused on the cytotoxicity of mitochondria-targeting antitumor drugs against cancer cells. The immune system activation potential of these drugs also needs to be explored in order to reprogram the immunosuppressive tumor microenvironment (TME).^13^

The immunosuppressive environment of solid tumors could be modulated by the innate and adaptive immune systems.^14,15^ As innate immune cells, macrophages are briefly divided into antitumor M1 phenotype and pro-tumor M2 phenotype based on the metabolic and functional differences.^16,17^ The status of mitochondria function have been reported to affect the metabolic and phenotypic reprogramming of macrophages.^18,19^ Moreover, stimulation of reactive oxygen species (ROS),^20−23^ cytokines,^24^ metabolites,^25^ and other signaling mediators^26^ also have been reported to actively reprogram macrophage metabolism and regulate its polarization.^27,28^ Thus, regulating mitochondrial ROS (mtROS) and mitochondrial metabolism to convert the tumor-associated macrophages (TAMs) toward the M1 phenotype for antitumor treatment is a promising approach.

ROS generation is the main antitumor mechanism for photodynamic therapy (PDT), among which type I PDT can help overcome the hypoxic tumor microenvironment due to its lower O_2_-dependence.^29,30^ Organelle-targeted photosensitizers are typically appreciated for their higher selectivity, lower side effects, and enhanced photodynamic efficacy.^31,32^ Meanwhile, metal complexes have been reported to disturb redox/energy/immune homeostasis in tumor cells to exert antitumor activity and mediate immune response, accompanied by different cell death pathways.^33−36^ In addition, immunogenic cell death (ICD), as a regulatory cell death pathway that activates adaptive immunity through the release of DAMPs, can also be induced by ROS overaccumulation.^37^

**Scheme 1:**
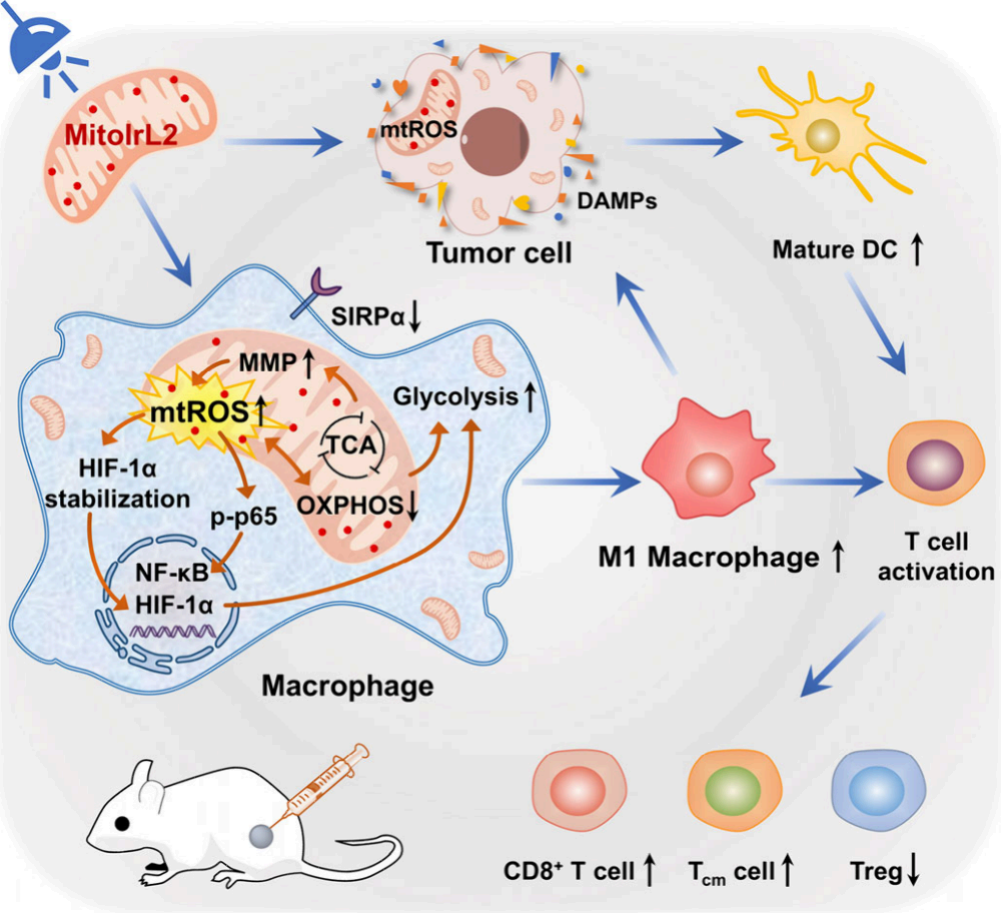
Schematic Illustration of Synergistic Mechanism Photo-Induced by **MitoIrL2** for Metalloimmunotherapy

Herein, we explored the mechanism underlying the synergistic antitumor immunity of macrophages and cancer cells triggered by the mitochondrial targeting iridium photosensitizer **MitoIrL2** (Scheme 1). On the one hand, the mitochondrial adjustment by photoinduced ROS drove the metabolic reprogramming from mitochondrial oxidative phosphorylation (OXPHOS) to glycolysis, thereby adjusting the macrophages into the antitumor M1 phenotype. Regulation of mitochondrial respiratory chain, HIF-1α stabilization, activation of NF-κB pathway, and reduction of SIRPα were successively demonstrated. At the same time, the photoinduced oxidative stress in mitochondria enhanced ICD in tumor cells. Moreover, the reversal of the immunosuppressive tumor microenvironment and establishment of long-term immune memory have also been illustrated *in vivo*. This work provides an effective strategy for simultaneous regulation of macrophages and tumor cells in the tumor microenvironment, which opened up a novel avenue for the metalloimmunotherapy.

## Results and Discussion

**Figure 1:**
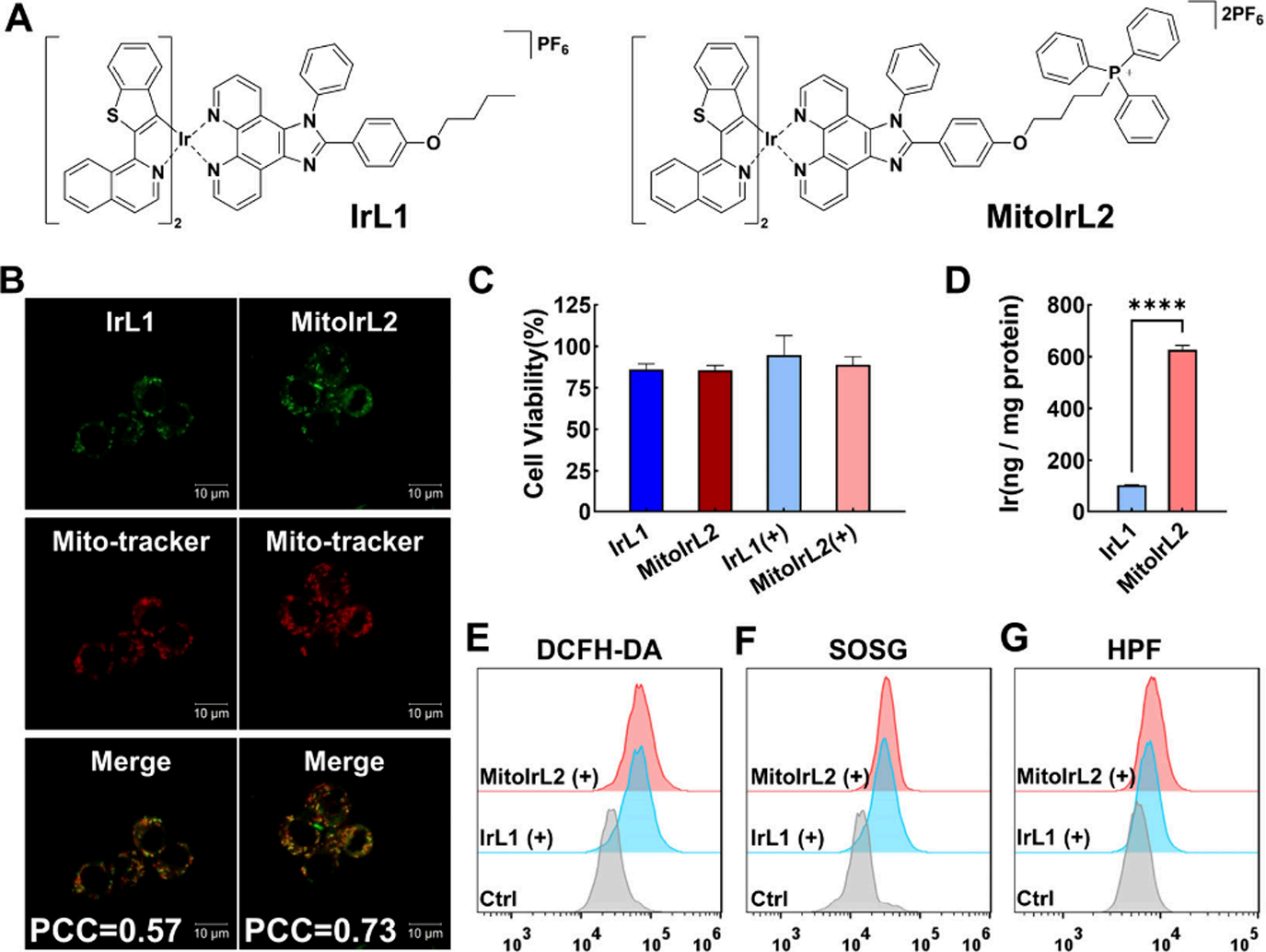
Basic characteristics of the two iridium complexes in RAW264.7 cells. (A) Chemical structures of **IrL1** and **MitoIrL2**. (B) Colocalization assay of two complexes (10 μM, λ_ex_ = 488 nm) with MitoTracker Deep Red (1 μM, λ_ex_ = 633 nm). Scale bars: 10 μm. (C) The 24 h cell viability of RAW264.7 cells after incubation with metal complexes (0.125 μM) in the absence and presence of light. (D) Iridium content in RAW264.7 cells determined by ICP-MS after 4 h of incubation with Ir(III) complexes (10 μM). Flow cytometry analysis of photoinduced ROS in RAW264.7 cells treated with Ir(III) complexes (0.1 μM) at different conditions using (E) DCFH-DA (10 μM), (F) SOSG (5 μM), and (G) HPF (10 μM). (+): white light irradiation after 4 h incubation, 6 mW/cm^2^, 15 min.

Metal complexes are widely used as potent photosensitizers (PSs), since their photophysical and photochemical properties could be easily regulated through the rich selection of metal centers, ligands, and coordination geometries.^38,39^ Covalent linkage of a lipophilic cationic triphenylphosphonium (TPP) group is one of the classical mitochondrial targeting methods.^40^ Previous studies have fully discussed the process of tumor cell death induced by Ir(III) based PSs through the ROS generation, especially the damages on mitochondrial morphology and function.^41−43^ In this study, **MitoIrL2**, a mitochondria-targeting iridium type I PS with a TPP group, was prepared according to the reported procedure, and **IrL1** without a TPP group was used as a control (Figure 1A).^43^ We focused on how photosensitizers regulate mitochondria to mediate the immune response in macrophages and tumor cells.

### Cell Uptake, Distribution, and ROS Generation

The mouse mononuclear macrophage leukemia cell line (RAW264.7) was selected as the primary model for macrophage studies *in vitro*. First, the subcellular accumulation of the two complexes was investigated by confocal imaging. As expected, **MitoIrL2** showed better overlap with commercial MitoTracker Deep Red, with a Pearson’s correlation coefficient (PCC) of 0.73 compared to 0.57 for **IrL1**, confirming the mitochondria targeting ability of the TPP group (Figure 1B). As shown in Figure 1C, both **MitoIrL2** and **IrL1** had no effect on cell viability at a concentration of 0.125 μM regardless of the irradiation. In addition, according to the inductively coupled plasma mass spectrometry (ICP-MS) measurement, the cell uptake of **MitoIrL2** was significantly higher than that of **IrL1** (Figure 1D). Therefore, we chose 0.1 μM as the safe incubation concentration for the subsequent experiments.

Next, we measured the ROS generation in RAW264.7 cells by flow cytometry to detect fluorescence changes of commercial sensors. 2′,7′-Dichlorodihydrofluorescein diacetate (DCFH-DA) is often used to investigate the total intracellular ROS production, while singlet oxygen sensor green (SOSG) and hydroxyphenyl fluorescein (HPF) are, respectively, used to detect ^1^O_2_ and OH^•−^. It can be seen that the intensities of DCFH-DA, SOSG, and HPF were enhanced distinctly after the incubation of the complexes (0.1 μM) and irradiation compared with the control, validating the increase of ROS generation (Figures 1E–1G).

### Photoinduced mtROS Triggered Macrophage M1-Like Polarization

**Figure 2:**
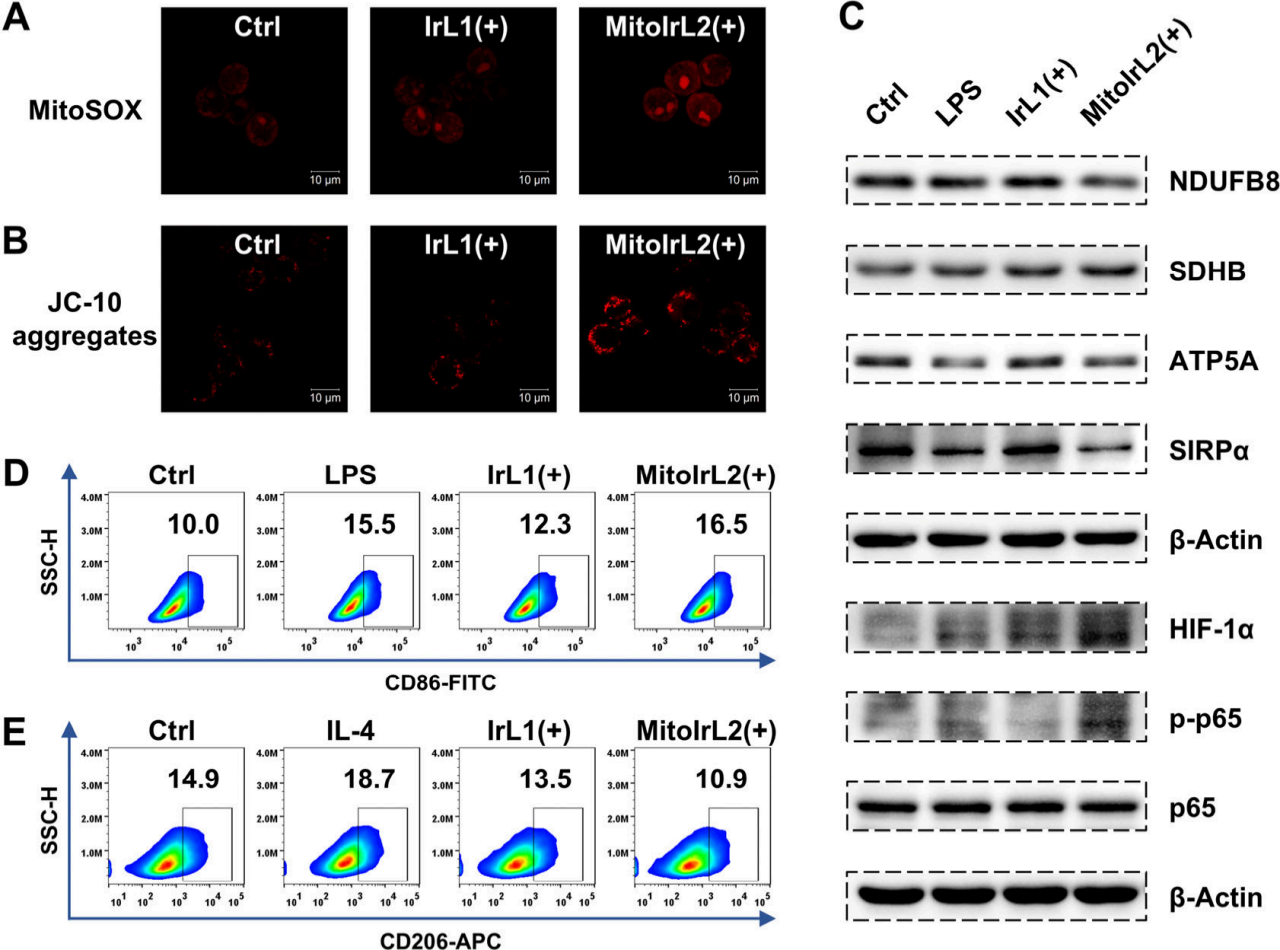
Confocal imaging of the (A) MitoSOX generation and the (B) JC-10 aggregates (λ_ex_ = 488 nm). Scale bars: 10 μm. (C) Western blot analysis of related protein expression in RAW264.7 cells treated with LPS (100 ng/mL) and the complexes (0.1 μM). Flow cytometry analysis of (D) CD86 and (E) CD206 expression in RAW264.7 cells after the 24 h treatment with LPS (100 ng/mL), IL-4 (20 ng/mL), and the complexes (0.1 μM). (+): white light irradiation after 4 h incubation, 6 mW/cm^2^, 15 min.

Then, the mtROS generation ability of **MitoIrL2** in RAW264.7 cells was evaluated. Mitochondria targeting MitoSOX Red was chosen as the mtROS indicator; it could be selectively oxidized by superoxide, which then binds nucleic acids to produce red fluorescence. Confocal imaging demonstrated mitochondria-targeted **MitoIrL2** could generate more MitoSOX than **IrL1** (Figure 2A). Mitochondrial membrane potential (MMP) is regarded as one of the important indexes to evaluate the normal function of mitochondria.^44,45^ We found enhanced fluorescence of JC-10 aggregates in RAW264.7 treated with **MitoIrL2** photoinduction, indicating an increase in MMP (Figure 2B). This phenomenon is similar to that of lipopolysaccharide (LPS)-activated metabolic reprogramming of macrophages to drive their pro-inflammatory phenotype.^25,46^

Both the elevation of MMP and the increased oxidation of succinate via succinate dehydrogenase (SDH) are thought to be required for mtROS production and hypoxia-inducible factor 1α (HIF-1α) stabilization to promote the pro-inflammatory state of macrophages.^46^ We then performed Western blot analysis of some representative mitochondrial respiratory chain (MRC) related proteins extracted from RAW264.7 cells treated with metal complexes and irradiated 2 h later (Figure 2C). The **MitoIrL2** group showed lower levels of MRC complex I subunit NDUFB8 and complex V subunit ATP5A compared with controls, with a slight increase in the B subunit of SDH (SDHB) of complex II. Actually, the production of ATP is catalyzed by complex V (ATP synthase), and the studies of mitochondrial metabolism of macrophages suggested that the deficiency of the complex I subunit lead to an enhanced M1 polarization.^47^ Meanwhile, since the complex across the mitochondrial inner membrane by the proton pump is no longer used by ATP synthases to produce ATP, inducing reverse electron transport, this provided an explanation for the higher MMP (Figure 2B).^46,48^ These results demonstrated that the mitochondrial regulation by **MitoIrL2** with irradiation did affect the mitochondrial metabolism and showed great potential of promoting the macrophage pro-inflammatory phenotype.

In addition, the upregulation of HIF-1α and phosphorylated p65 were observed, indicating that ROS accumulation regulated the HIF-1α and nuclear factor κB (NF-κB) pathway (Figure 2C).^49−52^ Signal regulatory protein α (SIRPα), a famous immune checkpoint, is involved in the regulation of NF-κB and has an inhibitory effect on innate immunity.^53−57^ The downregulation of SIRPα revealed that **MitoIrL2** has the potential to block immune escape and break the immunosuppression of the tumor microenvironment (Figure 2C).

We next verified the phenotype of RAW264.7 cells treated with metal complexes and irradiated 24 h later by flow cytometry, using CD86 as the M1 macrophage marker and CD206 as the M2 macrophage marker. The positive control groups were incubated with LPS and IL-4, respectively. As shown in Figures 2D and 2E, the expression of CD86 on RAW264.7 cells treated with **MitoIrL2** was increased more compared with **IrL1** and the control group, while CD206 decreased evidently, indicating that mitochondria-targeted complex **MitoIrL2** could drive the polarization of macrophages more toward the antitumor M1 phenotype.

### Metabolic Transition from OXPHOS to Glycolysis

**Figure 3:**
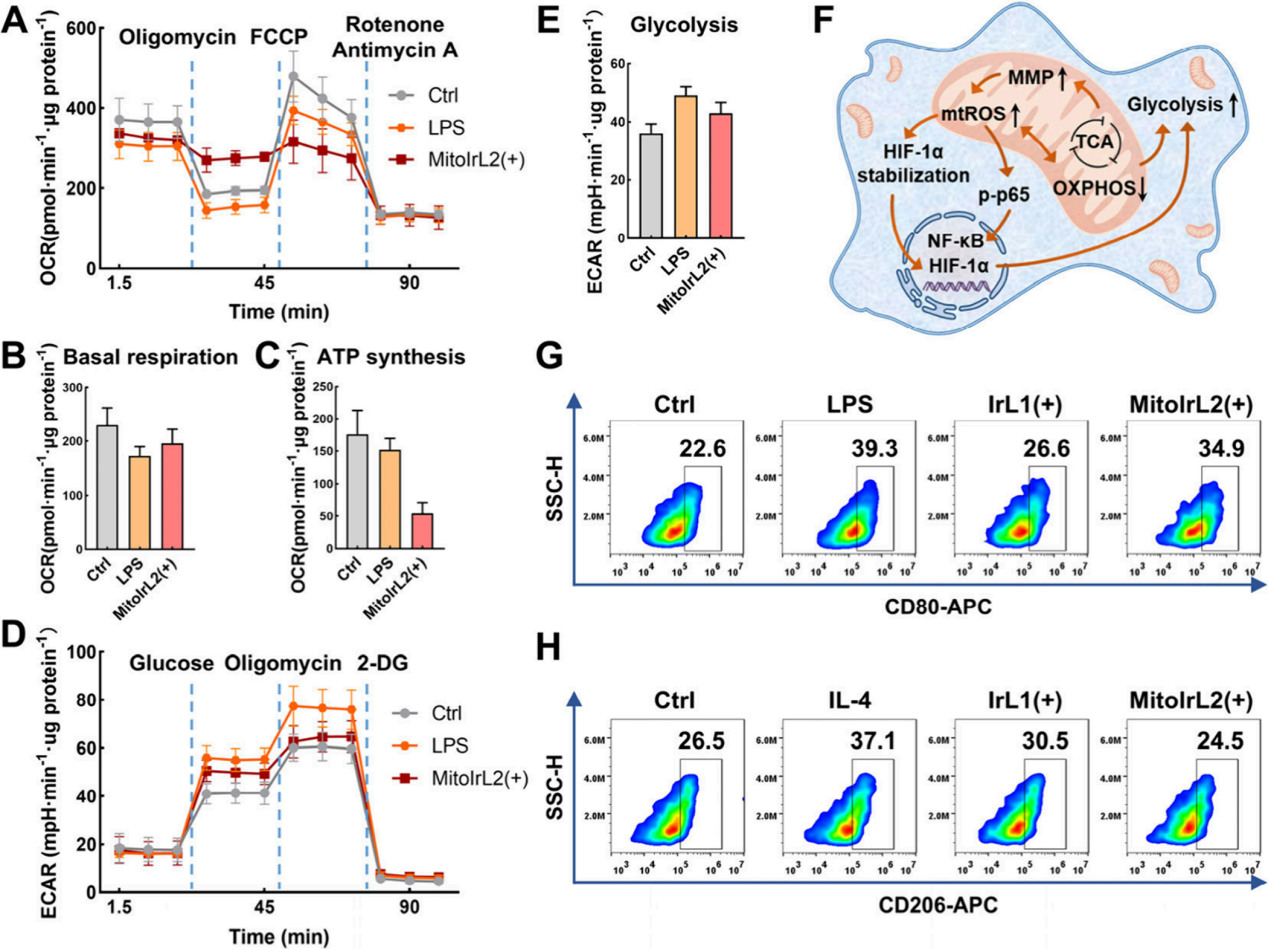
(A) OCR variations and quantification of (B) basal respiration and (C) ATP synthesis in RAW264.7 cells after treatment with LPS (100 ng/mL) or **MitoIrL2** (0.1 μM). (D) ECAR variations and (E) quantification of basal glycolysis in RAW264.7 cells after treatment with LPS (100 ng/mL) or **MitoIrL2** (0.1 μM). (F) Proposed mechanism of metabolic reprogramming in macrophages induced by **MitoIrL2**. Flow cytometry analysis of (G) CD80 and (H) CD206 expression in BMDMs after the 24 h treatment with LPS (100 ng/mL), IL-4 (20 ng/mL), and the complexes (0.1 μM). (+): white light irradiation after 4 h incubation, 6 mW/cm^2^, 15 min.

Due to the explosive energy requirement of rapid activation at the inflammatory site,^58^ the M1-like macrophages mainly utilize glycolytic metabolism, while the OXPHOS ability is decreased and the tricarboxylic acid cycle (TCA cycle) is blocked.^59^ The MRC deficiency and increased HIF-1α expression indicated that macrophages regulated by **MitoIrL2** with irradiation favored a glycolytic metabolic profile with the decreased OXPHOS capacity and broken TCA despite being in normoxic condition. To verify this inference, we measured a series of Seahorse assays by Agilent Seahorse XFe24 analyzer to assess mitochondrial and glycolytic function.^60^ RAW264.7 cells were tested after incubation and irradiation 2 h later with LPS as a reference. The cellular oxygen consumption rate (OCR) was evaluated by sequentially adding MRC-targeting inhibitors to reflect the capacity of mitochondrial OXPHOS. The oligomycin was an ATP synthase inhibitor, and carbonyl cyanide *p*-trifluoromethoxy phenylhydrazone (FCCP) was an uncoupler of the proton gradient across the inner mitochondrial membrane, while rotenone and antimycin A can inhibit the activity of complex I and complex III, respectively, thereby decreasing the level of the OCR to the minimum. Compared with the control group, **MitoIrL2** with irradiation induced a slight decrease in basal respiration, a distinct drop in ATP synthesis, and maximal respiration (Figures 3A–3C and Figure S1). Given the dynamic interaction between mitochondrial respiration and glycolysis, the extracellular acidification rate (ECAR) was measured. Since the cells were incubated in a test solution free of glucose and pyruvate, the basal glycolysis was evaluated by adding glucose, whereas 2-deoxyglucose (2-DG) was added to inhibit glycolysis by competitively binding to hexokinase in the glycolytic pathway. Macrophages induced by **MitoIrL2** with irradiation showed increased basal glycolysis and glycolytic capacity (Figures 3D and 3E and Figure S2). These results demonstrated the metabolic reprogramming of macrophages from the level of OXPHOS to glycolysis after **MitoIrL2** photoinduction (Figure 3F).

Bone marrow-derived macrophages (BMDMs), as primary macrophage cells, exhibit physiological features and functions more similar to those of macrophages *in vivo*. The enhancement of M1 macrophages and the decrease of M2 macrophages in BMDMs induced by **MitoIrL2** with irradiation were consistent with the results in the RAW264.7 cells (Figures 3G and 3H). Flow cytometry analysis confirmed the downregulation of SIRPα on BMDMs after **MitoIrL2** photoinduction as well (Figure S3). The data above sufficiently indicated that mitochondria-targeting complex **MitoIrL2** activated the pro-inflammatory phenotype of macrophages through metabolic reprogramming by regulating mitochondria, which is promising to break the immunosuppression of the tumor microenvironment.

### Mitochondria Targeting Enhanced 4T1 Cell Immunogenic Cell Death

**Figure 4:**
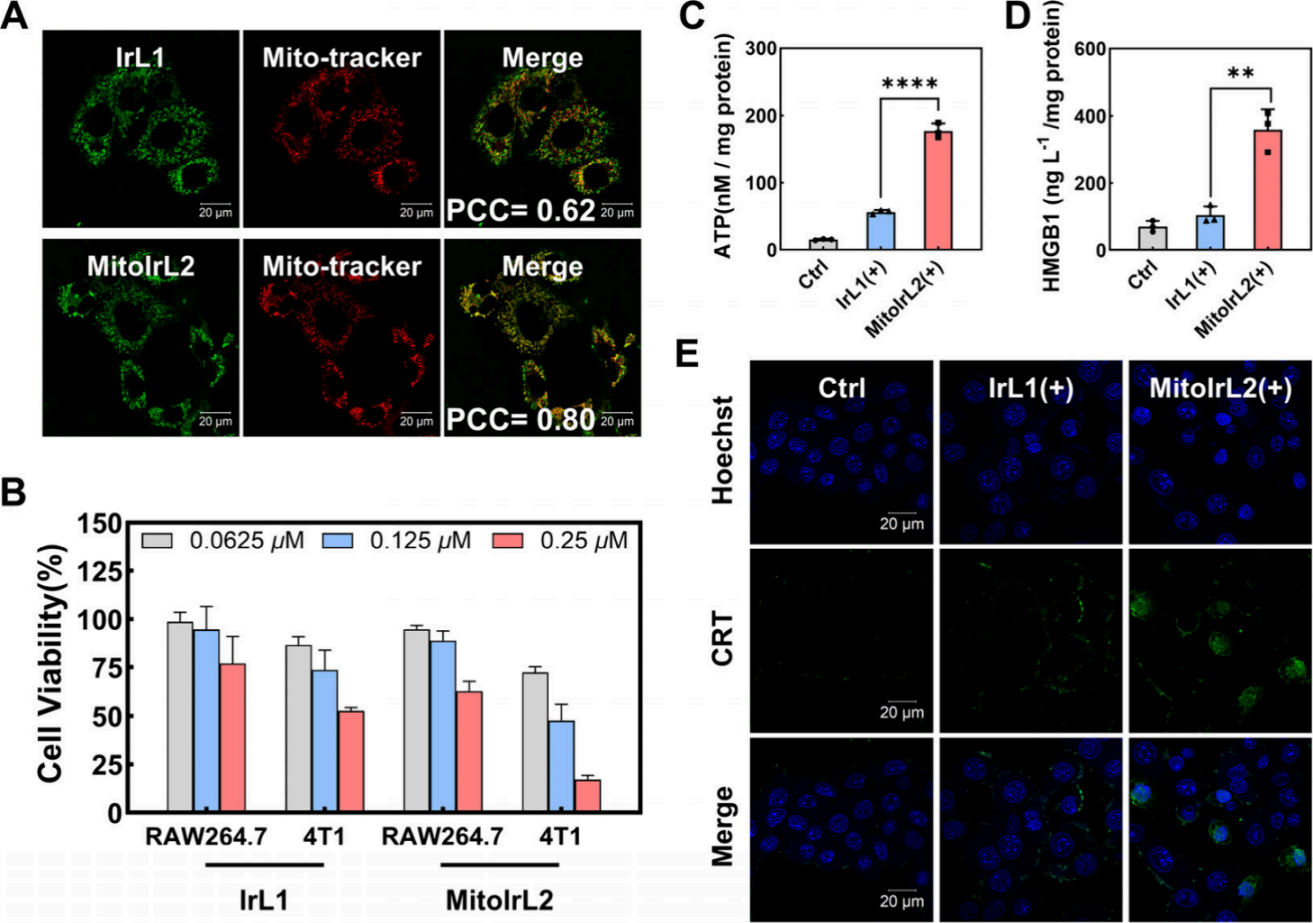
(A) Colocalization assay of two complexes (10 μM, λ_ex_ = 488 nm) with MitoTracker Deep Red (1 μM, λ_ex_ = 633 nm) in 4T1 cells. Scale bars: 20 μm. (B) The photocytotoxicity was 24 h for RAW264.7 and 4T1 cells after incubation with different concentrations. Extracellular release of (C) ATP and (D) HMGB1 in 4T1 cell culture supernatants. (E) Confocal imaging of the CRT exposure. The 4T1 cells were pretreated with complexes (0.1 μM). (+): white light irradiation after 4 h incubation, 6 mW/cm^2^, 15 min. Scale bars: 20 μm.

The mouse breast cancer cell line (4T1) was selected as the primary model for the tumor cell studies. **MitoIrL2** maintained good mitochondrial targeting in 4T1 cells, with confocal imaging shown in Figure 4A. **MitoIrL2** exhibited a higher photocytotoxicity than **IrL1**, with a half-maximal inhibitory concentration (IC_50_) of 0.11 ± 0.02 μM (Table S1). Comparing the viability of RAW264.7 and 4T1 cells after photoinduction, it was obvious that the living macrophages in the **MitoIrL2** group were more than 80% at 0.125 μM concentration, while half of the 4T1 cells died (Figure 4B). The survival of immune cells and the effective killing of tumor cells are the basis for the synergistic effect of drugs in the tumor microenvironment. ICP-MS analysis showed that the toxicity diversity between the two complexes was due to the difference in the cell uptake (Figure S4). Moreover, the photosensitizer **MitoIrL2** reduced MMP and damaged 4T1 cells by producing ROS (Figures S5–S7).

In the process of ICD, tumor cells release DAMPs with an “eat me” signal to promote the maturation of dendritic cells (DCs) and activate T cells, thus initiating the adaptive immunity.^61^ The ICD enhancement could be mediated by mitochondrial oxidative stress^62^ and ferroptosis.^36,63^ As shown in Figures 4C–4E, 4T1 cells induced by **MitoIrL2** with irradiation were detected to release more extracellular ATP and high mobility group box 1 (HMGB1), while the immunofluorescence imaging showed increased exposure of calreticulin (CRT) to the cell surface. The mitochondria-targeting complex did have the ability to enhance immunogenic cell death, thereby mediating the synergistic immune response from macrophages and cancer cells.

### *In Vivo* Imaging and Photodynamic Therapeutic Effects

**Figure 5:**
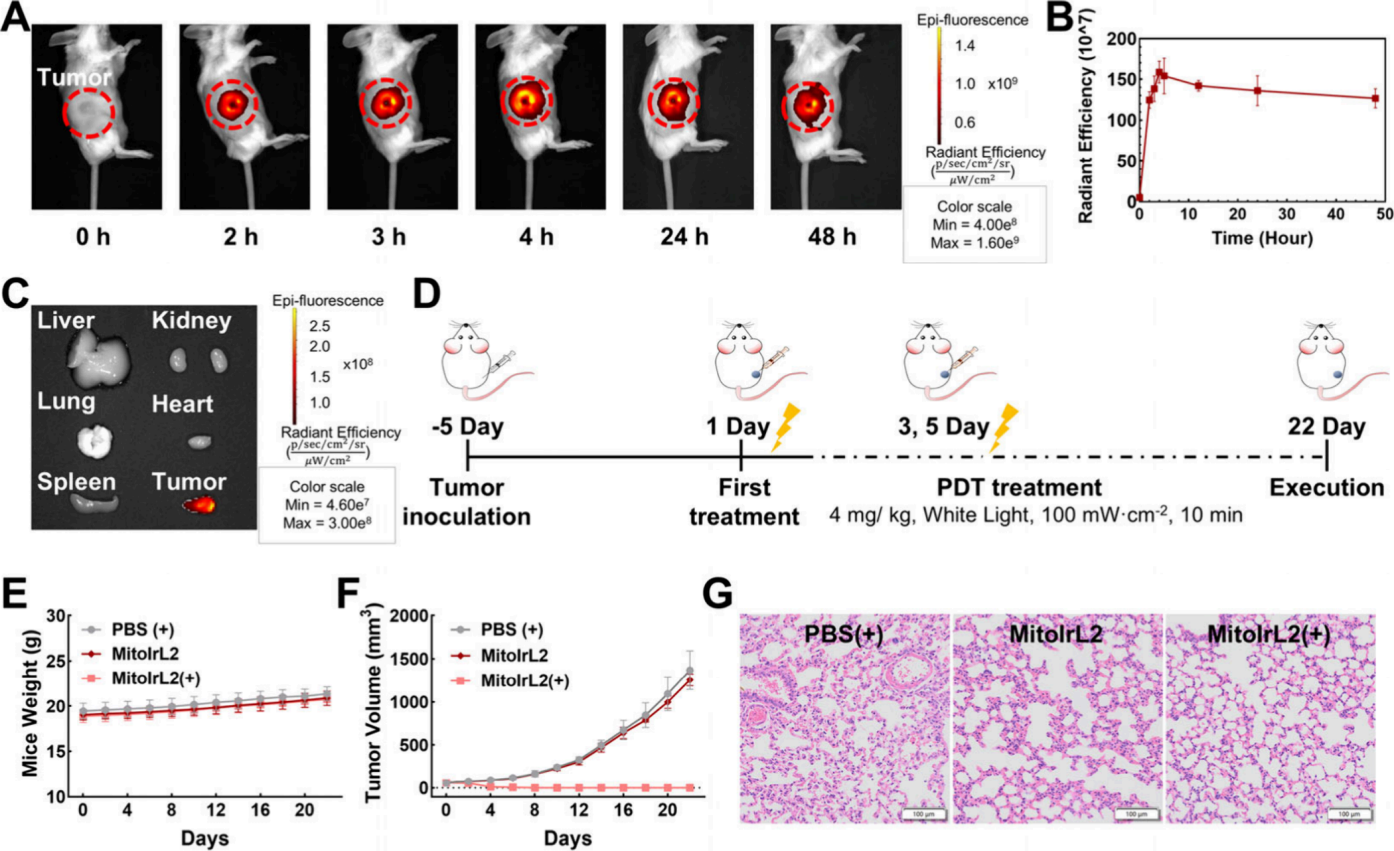
(A) Fluorescence imaging of 4T1 tumor-bearing mice after intratumoral injection with **MitoIrL2** (λ_ex_ = 500 nm, λ_em_ = 710 nm). (B) Fluorescence intensity of **MitoIrL2** in the tumor at different time points. (C) Fluorescence imaging of mouse organs collected 48 h postinjection. (D) Schematic illustration of the mouse model construction and therapy process. (E) Weight curves and (F) tumor volume of the mice with different treatments (*n* = 6). (G) H&E staining of lung tissue. Scale bars: 100 μm. **MitoIrL2**: 4 mg/kg. (+): white light irradiation after 4 h of incubation, 100 mW/cm^2^, 10 min.

In order to explore the antitumor potential of **MitoIrL2***in vivo*, 4T1 tumor-bearing BALB/c mice were constructed by inoculating 4T1 cells to the mice as primary tumors. The metabolism and distribution of the complex *in vivo* was first monitored using a PerkinElmer IVIS Lumina III *in vivo* imaging system. After intratumoral injection, **MitoIrL2** peaked at the tumor region at 4 h, then gradually diminished and remained mainly at the tumor site 48 h later, which was conducive to reducing side effects (Figures 5A–5C).

The antitumor efficiency *in vivo* was next evaluated. The mice were measured every 2 days and divided into three groups, PBS (+) and **MitoIrL2** and **MitoIrL2** (+) (Figure 5D). The body weight of the mice was consistently within the normal range compared to the control groups (Figure 5E). As shown in Figure 5F, the tumor disappeared in the **MitoIrL2** (+) group after three treatments. Notably, there was no sign of tumor recurrence in this group until the 22nd day, whereas the tumor volume in the PBS (+) and **MitoIrL2** treatment groups continued to increase at a similar rate (Figure 5F and Figure S8). In addition, the hematoxylin and eosin (H&E) staining of mouse lung tissues in each group confirmed the absence of metastasis (Figure 5G). These results indicated that **MitoIrL2** had good photodynamic efficacy and the potential to activate systemic immunity.

### Immunotherapy Effect Evaluation *In Vivo*

**Figure 6:**
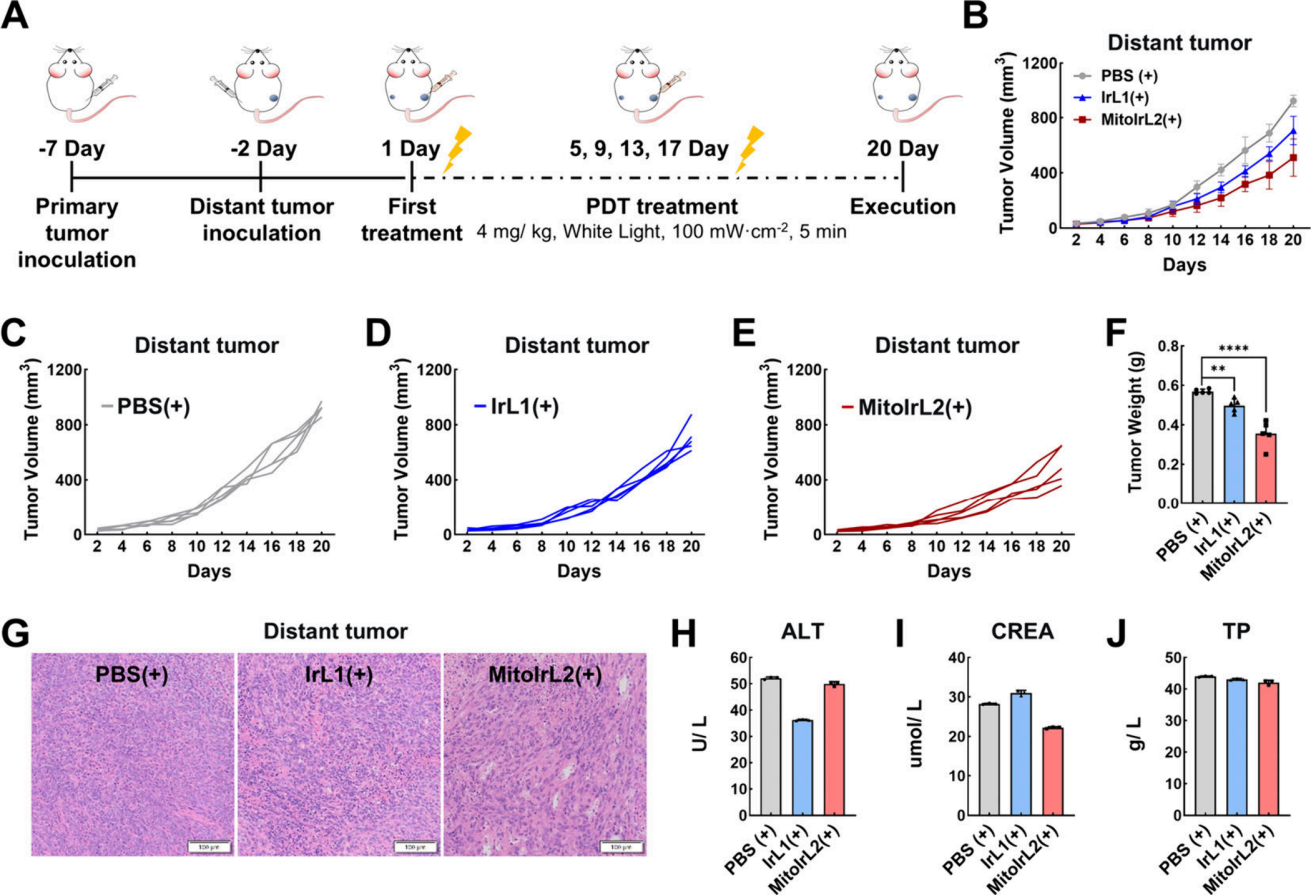
(A) Schematic illustration of the bilateral mouse model construction and therapy process. (B–E) Tumor volume curves of the mice with different treatments (*n* = 5). (F) Weight histogram of tumors after different treatments (*n* = 5). (G) H&E staining of distant tumor. Scale bars: 100 μm. (H–J) Blood biochemical analysis of the mice with different treatments (*n* = 3). **IrL1**, **MitoIrL2**: 4 mg/kg. (+): white light irradiation after 4 h incubation, 100 mW/cm^2^, 5 min.

Then, the bilateral 4T1 tumor-bearing mouse model was constructed to estimate the systemic immunity response mediated by **MitoIrL2**. As shown in Figure 6A, 4T1 cells were injected subcutaneously into the contralateral side as distant tumors 5 days after the primary tumor inoculation. The mice were divided into three groups, PBS (+), **IrL1** (+), and **MitoIrL2** (+). Intratumoral injection and irradiation was used to treat the primary tumors every 4 days, while distant tumors without any treatment were continuously monitored. The frequency of treatment and the duration of irradiation were halved compared to previous experiments *in vivo* to observe long-term responses. In contrast with the PBS (+) group, the growth of the distant tumor in the **MitoIrL2** (+) group was effectively suppressed, and the inhibition rate reached 40% at the end of the treatment (Figures 6B–6F). The damage of the primary and distant tumors demonstrated the photodynamic and immunologic efficacy of **MitoIrL2**, while the good condition of the organ tissues and the normal body weight curves attested to the absence of obvious systemic toxicity (Figure 6G and Figures S9 and S10).

Moreover, the blood biochemical indexes were all evaluated, including alanine transaminase (ALT), aspartate transaminase (AST), total bilirubin (T-BIL), creatinine (CREA), blood urea nitrogen (BUN), and serum total protein (TP), which were related to the important liver and kidney function (Figures 6H–6J and Figure S11). Meanwhile, the standard hematology markers, such as red blood cells (RBC), white blood cells (WBC), hematocrit (HCT), hemoglobin (HGB), mean corpuscular hemoglobin (MCH), and mean corpuscular volume (MCV), were assessed as well (Figure S12). The blood indexes mentioned above in the **MitoIrL2** (+) group were all within the normal range, indicating the excellent biosafety of **MitoIrL2**.

**Figure 7:**
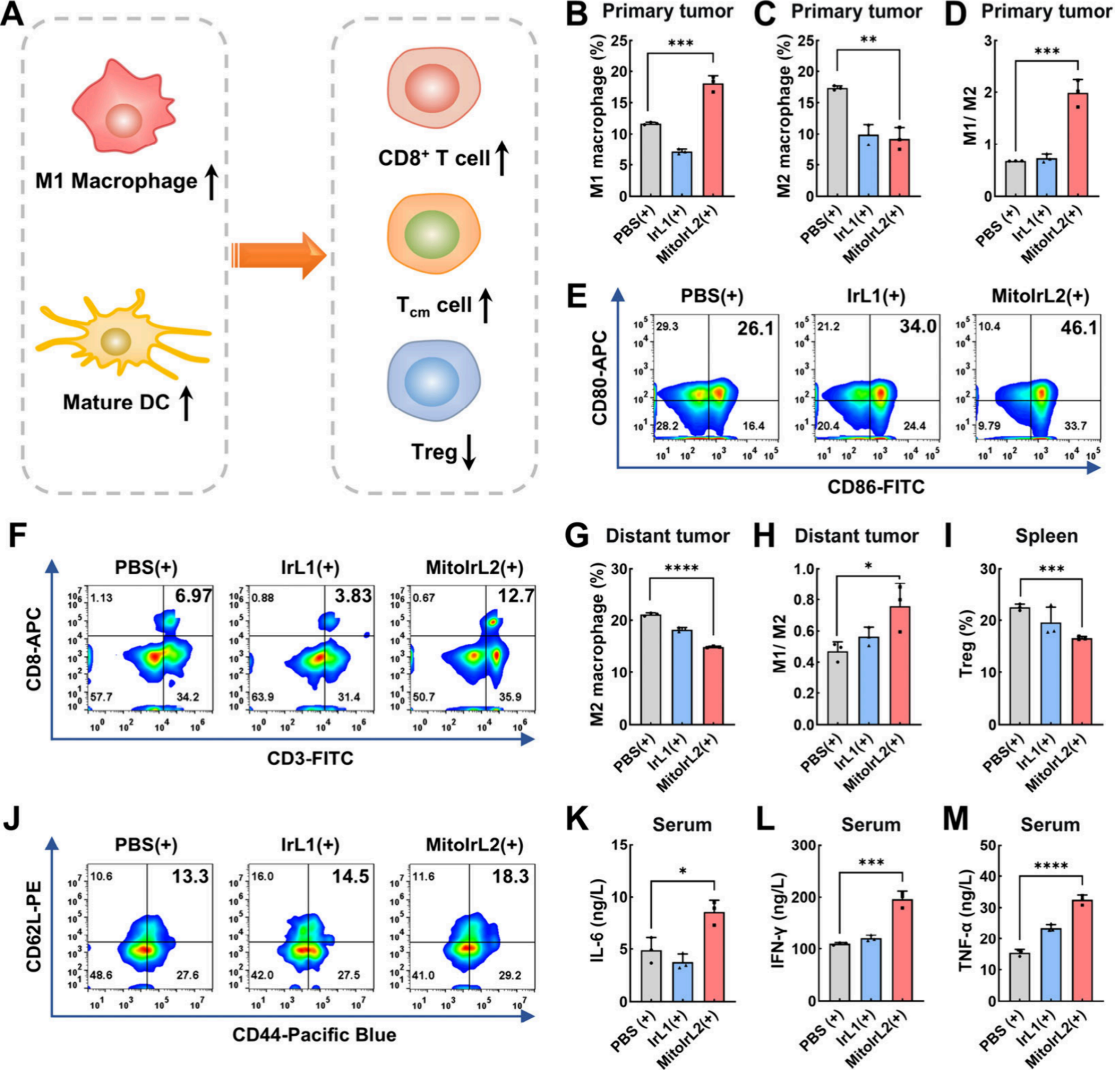
(A) Schematic illustration of the immune response *in vivo*. Flow cytometry analysis of (B) M1 macrophages, (C) M2 macrophages, (D) the ratio of M1/M2 macrophages, and (F) CD3^+^CD8^+^ T cells in primary tumor. (E) Flow cytometry analysis of DCs maturation in lymph nodes. Quantitative analysis of (G) the proportion of M2 macrophages and (H) the ratio of M1/M2 macrophages in distant tumor. Flow cytometry analysis of (I) Tregs and (J) central memory T cells in spleens. The cytokine secretion level of (K) IL-6, (L) IFN-γ, and (M) TNF-α in serum. All data presented as mean ± SD (*n* = 3). *p < 0.05, **p < 0.01, ***p < 0.001, ****p < 0.0001. **IrL1**, **MitoIrL2**: 4 mg/kg. (+): white light irradiation after 4 h incubation, 100 mW/cm^2^, 5 min.

To further evaluate the immunostimulatory efficiency *in vivo*, tumors, lymph nodes, and spleens after 8 days of treatment were extracted into a single-cell suspension, then stained with various relevant antibodies and analyzed by flow cytometry. Considering that modulating macrophage M1 polarization is an effective strategy to reverse immunosuppressive “cold” tumors, phenotypic changes of macrophages in primary tumors were first investigated. Compared with the PBS (+) group, M1 macrophages experienced a significant increase in the **MitoIrL2** (+) group (11.7% to 19.1%), while the proportion of M2 macrophages decreased from 17.3% to 7.49% (Figures 7B and 7C and Figures S13 and S14). Obviously, the ratio of M1 to M2 macrophages in the primary tumors photoinduced with the mitochondrial-targeted PS **MitoIrL2** was nearly 3-fold higher than in the other two groups (Figure 7D), revealing that **MitoIrL2** effectively transformed the “cold” tumor into “hot” tumor. Besides, the flow cytometric results showed the percentage of DC maturation in lymph nodes of the **MitoIrL2** (+) group was higher than the PBS (+) group as well (46.1% to 26.1%) (Figure 7E). The cytotoxic T lymphocytes (CTLs) in tumors play a vital role in the immunological process. CD3^+^CD8^+^ T cells in primary tumors were then evaluated with a distinct increase (12.7% to 6.97%), thereby indicating immune activation at the administration site (Figure 7F).

Given that distal tumors reflect systemic activation of host immunity, the same flow cytometry assays were analyzed. The decreased proportion of M2 macrophages and increased infiltration of CD3^+^CD8^+^ T cells in distant tumors indicated that **MitoIrL2** had the ability to activate systemic antitumor immunity (Figures 7G and 7H and Figures S15–S17). Regulatory T cells (Tregs) as suppressive immune cells could also be observed with a certain reduction (Figure 7I and Figure S18). The content of central memory T cells (*T*_cm_) in the spleens was evaluated at the end of the treatment. The noticeable increase from 13.3% to 18.3% was observed, demonstrating the establishment of long-term immune memory (Figure 7J and Figure S19). In addition, the secretion of pro-inflammatory cytokines in serum was measured by ELISA assay. As shown in Figures 7K–7M, the secretion level of IL-6, IFN-γ, and TNF-α in the **MitoIrL2** (+) group was considerably higher, thus revealing the strongest immune response. All of the data above demonstrated that **MitoIrL2** could reverse immunosuppressive tumor microenvironment and establish long-term immunological memory.

## Conclusion

In summary, a mitochondria-targeted iridium complex **MitoIrL2** has been investigated to trigger synergistic immune response in macrophages and tumor cells via photoinduced mitochondrial regulation. **MitoIrL2** has a higher phototoxicity to tumor cells than to macrophages, which is due to the cell uptake difference. The generation of mtROS was found to affect the mitochondrial respiratory chain, upregulate HIF-1α expression, and activate the NF-κB pathway in RAW264.7 cells, which synergistically drove the metabolic reprogramming from OXPHOS to glycolysis, thereby promoting macrophage polarization into the antitumor M1 phenotype. In addition, the expression of immune checkpoint SIRPα was downregulated on macrophages, which is conducive to breaking the immunosuppression of the tumor microenvironment. Meanwhile, mitochondrial targeting of the photosensitizer also enhanced its ability to induce ICD in 4T1 cells. **MitoIrL2** showed decent photodynamic therapeutic efficacy *in vivo* and effectively activated a systemic immunity response with good biosafety. The increased proportion of M1 macrophages in primary and distant tumors, along with the DC maturation, coenhanced the infiltration of CD3^+^CD8^+^ T cells. Moreover, the decrease of Tregs and the increase of *T*_cm_ cells illustrated that **MitoIrL2** could not only reverse immunosuppressive tumor microenvironment but also establish long-term immunological memory. This study opens up a promising strategy for metalloimmunotherapy to regulate the complicated tumor microenvironment.

## Supporting Information Available

Additional details including reagents, materials, instruments, and experimental procedures, as well as the supplementary figures and tables (PDF)

## References

[ref1] Chandel N. S. Evolution of Mitochondria as Signaling Organelles. Cell Metab. 2015, 22 (2), 204–206. 10.1016/j.cmet.2015.05.013. 26073494

[ref2] Suomalainen A.; Nunnari J. Mitochondria at the crossroads of health and disease. Cell 2024, 187 (11), 2601–2627. 10.1016/j.cell.2024.04.037. 38788685

[ref3] Bock F. J.; Tait S. W. G. Mitochondria as multifaceted regulators of cell death. Nat. Rev. Mol. Cell Biol. 2020, 21 (2), 85–100. 10.1038/s41580-019-0173-8. 31636403

[ref4] Wallace D. C. Mitochondria and cancer. Nat. Rev. Cancer 2012, 12 (10), 685–698. 10.1038/nrc3365. 23001348 PMC4371788

[ref5] Pagliarini D. J.; Rutter J. Hallmarks of a new era in mitochondrial biochemistry. Genes Dev. 2013, 27 (24), 2615–2627. 10.1101/gad.229724.113. 24352419 PMC3877752

[ref6] Guo X. L.; Yang N. D.; Ji W. H.; Zhang H.; Dong X.; Zhou Z. Q.; Li L.; Shen H. M.; Yao S. Q.; Huang W. Mito-Bomb: Targeting Mitochondria for Cancer Therapy. Adv. Mater. 2021, 33 (43), 202007778 10.1002/adma.202170340. 34510563

[ref7] Wang S. S.; Gai L. Z.; Chen Y. C.; Ji X. B.; Lu H.; Guo Z. J. Mitochondria-targeted BODIPY dyes for small molecule recognition, bio-imaging and photodynamic therapy. Chem. Soc. Rev. 2024, 53 (8), 3976–4019. 10.1039/D3CS00456B. 38450547

[ref8] Jin P.; Jiang J. W.; Zhou L.; Huang Z.; Nice E. C.; Huang C. H.; Fu L. Mitochondrial adaptation in cancer drug resistance: prevalence, mechanisms, and management. J. Hematol. Oncol. 2022, 15 (1), 97 10.1186/s13045-022-01313-4. 35851420 PMC9290242

[ref9] Weinberg S. E.; Sena L. A.; Chandel N. S. Mitochondria in the Regulation of Innate and Adaptive Immunity. Immunity 2015, 42 (3), 406–417. 10.1016/j.immuni.2015.02.002. 25786173 PMC4365295

[ref10] Mehta M. M.; Weinberg S. E.; Chandel N. S. Mitochondrial control of immunity: beyond ATP. Nat. Rev. Immunol. 2017, 17 (10), 608–620. 10.1038/nri.2017.66. 28669986

[ref11] Marchi S.; Guilbaud E.; Tait S. W. G.; Yamazaki T.; Galluzzi L. Mitochondrial control of inflammation. Nat. Rev. Immunol. 2023, 23 (3), 159–173. 10.1038/s41577-022-00760-x. 35879417 PMC9310369

[ref12] Martínez-Reyes I.; Chandel N. S. Mitochondrial TCA cycle metabolites control physiology and disease. Nat. Commun. 2020, 11 (1), 102 10.1038/s41467-019-13668-3. 31900386 PMC6941980

[ref13] Binnewies M.; Roberts E. W.; Kersten K.; Chan V.; Fearon D. F.; Merad M.; Coussens L. M.; Gabrilovich D. I.; Ostrand-Rosenberg S.; Hedrick C. C.; et al. Understanding the tumor immune microenvironment (TIME) for effective therapy. Nat. Med. 2018, 24 (5), 541–550. 10.1038/s41591-018-0014-x. 29686425 PMC5998822

[ref14] Arner E. N.; Rathmell J. C. Metabolic programming and immune suppression in the tumor microenvironment. Cancer Cell 2023, 41 (3), 421–433. 10.1016/j.ccell.2023.01.009. 36801000 PMC10023409

[ref15] Bader J. E.; Voss K.; Rathmell J. C. Targeting Metabolism to Improve the Tumor Microenvironment for Cancer Immunotherapy. Mol. Cell 2020, 78 (6), 1019–1033. 10.1016/j.molcel.2020.05.034. 32559423 PMC7339967

[ref16] Murray P. J. Macrophage Polarization. Annu. Rev. Physiol. 2017, 79, 541–566. 10.1146/annurev-physiol-022516-034339. 27813830

[ref17] Bronte V.; Murray P. J. Understanding Local Macrophage Phenotypes In Disease: Modulating macrophage function to treat cancer. Nat. Med. 2015, 21 (2), 117–119. 10.1038/nm.3794. 25654601

[ref18] Formentini L.; Santacatterina F.; Nunez de Arenas C.; Stamatakis K.; Lopez-Martinez D.; Logan A.; Fresno M.; Smits R.; Murphy M. P.; Cuezva J. M. Mitochondrial ROS Production Protects the Intestine from Inflammation through Functional M2Macrophage Polarization. Cell Rep. 2017, 19 (6), 1202–1213. 10.1016/j.celrep.2017.04.036. 28494869

[ref19] Willenborg S.; Sanin D. E.; Jais A.; Ding X. L.; Ulas T.; Nüchel J.; Popovic M.; MacVicar T.; Langer T.; Schultze J. L.; et al. Mitochondrial metabolism coordinates stage-specific repair processes in macrophages during wound healing. Cell Metab. 2021, 33 (12), 2398–2414. 10.1016/j.cmet.2021.10.004. 34715039

[ref20] Zhou Y.; Que K. T.; Zhang Z.; Yi Z. J.; Zhao P. X.; You Y.; Gong J. P.; Liu Z. J. Iron overloaded polarizes macrophage to proinflammation phenotype through ROS/acetyl-p53 pathway. Cancer Med. 2018, 7 (8), 4012–4022. 10.1002/cam4.1670. 29989329 PMC6089144

[ref21] Tan H. Y.; Wang N.; Li S.; Hong M.; Wang X. B.; Feng Y. B. The Reactive Oxygen Species in Macrophage Polarization: Reflecting Its Dual Role in Progression and Treatment of Human Diseases. Oxid. Med. Cell. Longev. 2016, 2016, 2795090 10.1155/2016/2795090. 27143992 PMC4837277

[ref22] Yang G.; Ni J. S.; Li Y. X.; Zha M. L.; Tu Y.; Li K. Acceptor Engineering for Optimized ROS Generation Facilitates Reprogramming Macrophages to M1 Phenotype in Photodynamic Immunotherapy. Angew. Chem., Int. Ed. 2021, 60 (10), 5386–5393. 10.1002/anie.202013228. 33236483

[ref23] West A. P.; Brodsky I. E.; Rahner C.; Woo D. K.; Erdjument-Bromage H.; Tempst P.; Walsh M. C.; Choi Y.; Shadel G. S.; Ghosh S. TLR signalling augments macrophage bactericidal activity through mitochondrial ROS. Nature 2011, 472 (7344), 476–480. 10.1038/nature09973. 21525932 PMC3460538

[ref24] Van den Bossche J.; Baardman J.; Otto N. A.; van der Velden S.; Neele A. E.; van den Berg S. M.; Luque-Martin R.; Chen H.-J.; Boshuizen M. C.S.; Ahmed M.; Hoeksema M. A.; de Vos A. F.; de Winther M. P.J.; et al. Mitochondrial Dysfunction Prevents Repolarization of Inflammatory Macrophages. Cell Rep. 2016, 17 (3), 684–696. 10.1016/j.celrep.2016.09.008. 27732846

[ref25] Tannahill G. M.; Curtis A. M.; Adamik J.; Palsson-McDermott E. M.; McGettrick A. F.; Goel G.; Frezza C.; Bernard N. J.; Kelly B.; Foley N. H.; et al. Succinate is an inflammatory signal that induces IL-1β through HIF-1α. Nature 2013, 496 (7444), 238–242. 10.1038/nature11986. 23535595 PMC4031686

[ref26] Liu P.-S.; Chen Y.-T.; Li X.; Hsueh P.-C.; Tzeng S.-F.; Chen H.; Shi P.-Z.; Xie X.; Parik S.; Planque M.; et al. CD40 signal rewires fatty acid and glutamine metabolism for stimulating macrophage anti-tumorigenic functions. Nat. Immunol. 2023, 24 (3), 452–462. 10.1038/s41590-023-01430-3. 36823405 PMC9977680

[ref27] Morris G.; Gevezova M.; Sarafian V.; Maes M. Redox regulation of the immune response. Cell. Mol. Immunol. 2022, 19 (10), 1079–1101. 10.1038/s41423-022-00902-0. 36056148 PMC9508259

[ref28] Mehla K.; Singh P. K. Metabolic Regulation of Macrophage Polarization in Cancer. Trends Cancer 2019, 5 (12), 822–834. 10.1016/j.trecan.2019.10.007. 31813459 PMC7187927

[ref29] Zhou Z. J.; Song J. B.; Nie L. M.; Chen X. Y. Reactive oxygen species generating systems meeting challenges of photodynamic cancer therapy. Chem. Soc. Rev. 2016, 45 (23), 6597–6626. 10.1039/C6CS00271D. 27722328 PMC5118097

[ref30] Wu Y. P.; Li S. M.; Chen Y. C.; He W. J.; Guo Z. J. Recent advances in noble metal complex based photodynamic therapy. Chem. Sci. 2022, 13 (18), 5085–5106. 10.1039/D1SC05478C. 35655575 PMC9093168

[ref31] Wang R.; Li X. S.; Yoon J. Organelle-Targeted Photosensitizers for Precision Photodynamic Therapy. ACS Appl. Mater. Interfaces 2021, 13 (17), 19543–19571. 10.1021/acsami.1c02019. 33900741

[ref32] Hong Luo G.; Zhao Xu T.; Li X.; Jiang W.; Hong Duo Y.; Zhong Tang B. Cellular organelle-targeted smart AIEgens in tumor detection, imaging and therapeutics. Coord. Chem. Rev. 2022, 462, 214508 10.1016/j.ccr.2022.214508.

[ref33] Englinger B.; Pirker C.; Heffeter P.; Terenzi A.; Kowol C. R.; Keppler B. K.; Berger W. Metal Drugs and the Anticancer Immune Response. Chem. Rev. 2019, 119 (2), 1519–1624. 10.1021/acs.chemrev.8b00396. 30489072

[ref34] Peng K.; Zheng Y.; Xia W.; Mao Z. W. Organometallic anti-tumor agents: targeting from biomolecules to dynamic bioprocesses. Chem. Soc. Rev. 2023, 52 (8), 2790–2832. 10.1039/D2CS00757F. 37014670

[ref35] Zhang Y. Y.; Doan B. T.; Gasser G. Metal-Based Photosensitizers as Inducers of Regulated Cell Death Mechanisms. Chem. Rev. 2023, 123 (16), 10135–10155. 10.1021/acs.chemrev.3c00161. 37534710

[ref36] Li S. M.; Yuan H.; Chen Y. C.; Guo Z. J. Metal complexes induced ferroptosis for anticancer therapy. Fundamental Res. 2023, 3 (4), 525–528. 10.1016/j.fmre.2022.10.001. PMC11197733 38933555

[ref37] Kroemer G.; Galluzzi L.; Kepp O.; Zitvogel L. Immunogenic Cell Death in Cancer Therapy. Annu. Rev. Immunol. 2013, 31, 51–72. 10.1146/annurev-immunol-032712-100008. 23157435

[ref38] Li Y. L.; Li A. J.; Huang S. L.; Vittal J. J.; Yang G. Y. Polypyridyl Ru(ii) or cyclometalated Ir(iii) functionalized architectures for photocatalysis. Chem. Soc. Rev. 2023, 52 (14), 4725–4754. 10.1039/D3CS00053B. 37382597

[ref39] Imberti C.; Zhang P. Y.; Huang H. Y.; Sadler P. J. New Designs for Phototherapeutic Transition Metal Complexes. Angew. Chem., Int. Ed. 2020, 59 (1), 61–73. 10.1002/anie.201905171. PMC6973108 31310436

[ref40] Zielonka J.; Joseph J.; Sikora A.; Hardy M.; Ouari O.; Vasquez-Vivar J.; Cheng G.; Lopez M.; Kalyanaraman B. Mitochondria-Targeted Triphenylphosphonium-Based Compounds: Syntheses, Mechanisms of Action, and Therapeutic and Diagnostic Applications. Chem. Rev. 2017, 117 (15), 10043–10120. 10.1021/acs.chemrev.7b00042. 28654243 PMC5611849

[ref41] Qiu K. Q.; Chen Y.; Rees T. W.; Ji L. N.; Chao H. Organelle-targeting metal complexes: From molecular design to bio-applications. Coord. Chem. Rev. 2019, 378, 66–86. 10.1016/j.ccr.2017.10.022.

[ref42] Kar B.; Das U.; Roy N.; Paira P. Recent advances on organelle specific Ru(II)/Ir(III)/Re(I) based complexes for photodynamic therapy. Coord. Chem. Rev. 2023, 474, 214860 10.1016/j.ccr.2022.214860.

[ref43] Yuan H.; Han Z.; Chen Y. C.; Qi F.; Fang H. B.; Guo Z. J.; Zhang S. R.; He W. J. Ferroptosis Photoinduced by New Cyclometalated Iridium(III) Complexes and Its Synergism with Apoptosis in Tumor Cell Inhibition. Angew. Chem., Int. Ed. 2021, 60 (15), 8174–8181. 10.1002/anie.202014959. 33656228

[ref44] Martínez-Reyes I.; Diebold L. P.; Kong H.; Schieber M.; Huang H.; Hensley C. T.; Mehta M. M.; Wang T.; Santos J. H.; Woychik R.; et al. TCA Cycle and Mitochondrial Membrane Potential Are Necessary for Diverse Biological Functions. Mol. Cell 2016, 61 (2), 199–209. 10.1016/j.molcel.2015.12.002. 26725009 PMC4724312

[ref45] Sanin D. E.; Matsushita M.; Klein Geltink R. I.; Grzes K. M.; Bakker N. V.; Corrado M.; Kabat A. M.; Buck M. D.; Qiu J.; Lawless S. J.; et al. Mitochondrial Membrane Potential Regulates Nuclear Gene Expression in Macrophages Exposed to Prostaglandin E2. Immunity 2018, 49 (6), 1021–1033. 10.1016/j.immuni.2018.10.011. 30566880 PMC7271981

[ref46] Mills E. L.; Kelly B.; Logan A.; Costa A. S. H.; Varma M.; Bryant C. E.; Tourlomousis P.; Däbritz J. H. M.; Gottlieb E.; Latorre I.; et al. Succinate Dehydrogenase Supports Metabolic Repurposing of Mitochondria to Drive Inflammatory Macrophages. Cell 2016, 167 (2), 457–470. 10.1016/j.cell.2016.08.064. 27667687 PMC5863951

[ref47] Jin Z. X.; Wei W.; Yang M.; Du Y.; Wan Y. H. Mitochondrial Complex I Activity Suppresses Inflammation and Enhances Bone Resorption by Shifting Macrophage-Osteoclast Polarization. Cell Metab. 2014, 20 (3), 483–498. 10.1016/j.cmet.2014.07.011. 25130399 PMC4156549

[ref48] Benmoussa K.; Garaude J.; Acín-Pérez R. How Mitochondrial Metabolism Contributes to Macrophage Phenotype and Functions. J. Mol. Biol. 2018, 430 (21), 3906–3921. 10.1016/j.jmb.2018.07.003. 30006265

[ref49] McGettrick A. F.; O’Neill L. A. J. The Role of HIF in Immunity and Inflammation. Cell Metab. 2020, 32 (4), 524–536. 10.1016/j.cmet.2020.08.002. 32853548

[ref50] Chen M. M.; Zhang Y.; Zhou P. H.; Liu X. Z.; Zhao H.; Zhou X. C.; Gu Q. L.; Li B.; Zhu X. S.; Shi Q. Substrate stiffness modulates bone marrow-derived macrophage polarization through NF-κB signaling pathway. Bioact. Mater. 2020, 5 (4), 880–890. 10.1016/j.bioactmat.2020.05.004. 32637751 PMC7332470

[ref51] Rendra E.; Riabov V.; Mossel D. M.; Sevastyanova T.; Harmsen M. C.; Kzhyshkowska J. Reactive oxygen species (ROS) in macrophage activation and function in diabetes. Immunobiology 2019, 224 (2), 242–253. 10.1016/j.imbio.2018.11.010. 30739804

[ref52] Nathan C.; Cunningham-Bussel A. Beyond oxidative stress: an immunologist’s guide to reactive oxygen species. Nat. Rev. Immunol. 2013, 13 (5), 349–361. 10.1038/nri3423. 23618831 PMC4250048

[ref53] Liu Y.; Wang Y. J.; Yang Y. R.; Weng L. J.; Wu Q.; Zhang J.; Zhao P. C.; Fang L.; Shi Y. F.; Wang P. Emerging phagocytosis checkpoints in cancer immunotherapy. Signal Transduct. Target. Ther. 2023, 8 (1), 104 10.1038/s41392-023-01365-z. 36882399 PMC9990587

[ref54] Kong X. N.; Yan H. X.; Chen L.; Dong L. W.; Yang W.; Liu Q.; Yu L. X.; Huang D. D.; Liu S. Q.; Liu H.; et al. LPS-induced down-regulation of signal regulatory protein α contributes to innate immune activation in macrophages. J. Exp. Med. 2007, 204 (11), 2719–2731. 10.1084/jem.20062611. 17954568 PMC2118489

[ref55] Veillette A.; Chen J. SIRPα-CD47 Immune Checkpoint Blockade in Anticancer Therapy. Trends Immunol. 2018, 39 (3), 173–184. 10.1016/j.it.2017.12.005. 29336991

[ref56] Willingham S. B.; Volkmer J. P.; Gentles A. J.; Sahoo D.; Dalerba P.; Mitra S. S.; Wang J.; Contreras-Trujillo H.; Martin R.; Cohen J. D.; et al. The CD47-signal regulatory protein alpha (SIRPa) interaction is a therapeutic target for human solid tumors. Proc. Natl. Acad. Sci. U.S.A. 2012, 109 (17), 6662–6667. 10.1073/pnas.1121623109. 22451913 PMC3340046

[ref57] Lian S.; Xie X.; Lu Y.; Lee J. Checkpoint CD47 Function On Tumor Metastasis And Immune Therapy. OncoTargets Ther. 2019, 12, 9105–9114. 10.2147/OTT.S220196. PMC6839575 31806995

[ref58] O’Neill L. A. J.; Pearce E. J. Immunometabolism governs dendritic cell and macrophage function. J. Exp. Med. 2016, 213 (1), 15–23. 10.1084/jem.20151570. 26694970 PMC4710204

[ref59] Russell D. G.; Huang L.; VanderVen B. C. Immunometabolism at the interface between macrophages and pathogens. Nat. Rev. Immunol. 2019, 19 (5), 291–304. 10.1038/s41577-019-0124-9. 30679807 PMC7032560

[ref60] Divakaruni A. S.; Paradyse A.; Ferrick D. A.; Murphy A. N.; Jastroch M. Analysis and Interpretation of Microplate-Based Oxygen Consumption and pH Data. Methods Enzymol. 2014, 547, 309–354. 10.1016/B978-0-12-801415-8.00016-3. 25416364

[ref61] Krysko D. V.; Garg A. D.; Kaczmarek A.; Krysko O.; Agostinis P.; Vandenabeele P. Immunogenic cell death and DAMPs in cancer therapy. Nat. Rev. Cancer 2012, 12 (12), 860–875. 10.1038/nrc3380. 23151605

[ref62] Chen C.; Ni X.; Jia S. R.; Liang Y.; Wu X. L.; Kong D. L.; Ding D. Massively Evoking Immunogenic Cell Death by Focused Mitochondrial Oxidative Stress using an AIE Luminogen with a Twisted Molecular Structure. Adv. Mater. 2019, 31 (52), e1904914 10.1002/adma.201904914. 31696981

[ref63] Wang W. J.; Ling Y. Y.; Zhong Y. M.; Li Z. Y.; Tan C. P.; Mao Z. W. Ferroptosis-Enhanced Cancer Immunity by a Ferrocene-Appended Iridium(III) Diphosphine Complex. Angew. Chem., Int. Ed. 2022, 61 (16), e202115247 10.1002/anie.202115247. 34965011

