# Supplementary material for: Synergistic Antitumor Immunotherapy via Mitochondria Regulation in Macrophages and Tumor Cells by an Iridium Photosensitizer
Source: ACS Cent Sci. 2025 Mar 11;11(3):441–51. doi: 10.1021/acscentsci.4c02156 (PMC11950858; doi:10.1021/acscentsci.4c02156)
Supplement: Supplementary file 1 — oc4c02156_si_001.pdf [file oc4c02156_si_001.pdf]

Supporting Information

Synergistic antitumor immunotherapy via mitochondria regulation in macrophages and tumor cells by an iridium photosensitizer

Shumeng Li[#], Hao Yuan[#], Xiu-Zhi Yang, Xinyu Xu, Wenhao Yu, Yanping Wu, Shankun Yao, Weijiang He, Zijian Guo*, Yuncong Chen*

Supporting Information

Contents

Experimental section 2

Materials and instruments 2
Cell culture 2
Confocal imaging 2
Cytotoxicity assay 3
Cell uptake 3
Flow cytometry analysis 3
Western blot analysis 3
Metabolic measurement 4
Detection of extracellular ATP and HMGB1 4
Breast cancer mouse model 4

Supporting figures 5

Figure S1. 5
Figure S2. 5
Figure S3. 6
Figure S4. 6
Figure S5. 7
Figure S6. 7
Figure S7. 7
Figure S8. 8
Figure S9. 8
Figure S10. 9
Figure S11. 9
Figure S12. 10
Figure S13. 10
Figure S14. 11
Figure S15. 11
Figure S16. 12
Figure S17. 12
Figure S18. 13
Figure S19. 13

Supporting table 14

Experimental section

Materials and instruments

All reagents were purchased commercially and used without further purification. Organelle tracker and ROS detection sensors were purchased from Thermo Fisher Scientific. NDUFB8 (ab192878), SDHB (ab175225), ATP5A (ab176569), SIRPα (ab191419), HIF-1α (ab179483), NF-κB p65 (ab32536), NF-κB p65 (phospho S536) (ab76302), Anti-Calreticulin (ab2907) were purchased from Abcam. β-Actin (#4970) was purchased from Cell Signaling Technology. FITC anti-mouse CD86 (11-0862-82), APC anti-mouse CD206 (17-2061-82), APC anti-mouse CD80 (17-0801-82), PE anti-mouse CD11c (12-0114-82), APC anti-mouse CD25 (17-0521-81), FITC anti-mouse Foxp3 (11-5773-82) were purchased from Thermo Fisher Scientific. FITC anti-mouse SIRPα (144006), PE anti-mouse CD4 (100512), FITC anti-mouse CD3 (100204), Pacific Blue anti-mouse CD44 (103020), PE anti-mouse CD62L (161204), FITC anti-mouse F4/80 (123107), Zombie Violet™ Fixable Viability Kit (423113), TruStain FcX™ PLUS (anti-mouse CD16/32) (156603) were purchased from BioLegend. APC anti-mouse CD8a (APC-65069), PE anti-mouse CD11b (PE-65055) were purchased from Proteintech Group. Enhanced ATP Assay Kit (S0027) was purchased from Beyotime. White LED was purchased from Shenzhen PURI materials technology co., LTD. Zeiss LSM-710 laser confocal microscope system was used for cell imaging, and PerkinElmer IVIS Lumina III *in vivo* imaging system performed imaging *in vivo*.

Cell culture

RAW264.7 and 4T1 cells were purchased from the American Type Culture Collection (ATCC). Culture media were purchased from Nanjing KeyGen Biotech Co., Ltd. including DMEM and RPMI-1640 medium. RAW264.7 cells were cultured in a humidified 37℃, 5% CO_2 incubator and maintained in DMEM medium supplemented with 10% fetal bovine serum (FBS) (Gibco), while 4T1 cells were cultured in RPMI-1640 medium containing 10% FBS (Hyclone).

Bone marrow-derived macrophages (BMDMs), which were obtained from bone marrow cells isolated from female C57BL/6 mice (4-6 weeks old), were cultured in DMEM medium containing 10% FBS (Gibco) and supplemented with 20 ng/mL macrophage colony-stimulating factor (M-CSF) for 6 days. The medium was replaced on day 3, and adherent BMDMs (M0-like) were harvested for experiments on day 6.

Confocal imaging

RAW264.7 and 4T1 cells were incubated with **MitoIrL2** or **IrL1** (10 μM, λ_{ex} = 488 nm, λ_{em} = 620-740 nm) at 37℃ for 4 h and then co-incubated with MitoTracker Deep Red (1 μM, λ_{ex} = 633 nm, λ_{em} = 650-750 nm) for 25 min at 37℃. Confocal imaging was performed after the entire incubation in the dark.

RAW264.7 and 4T1 cells were incubated with **MitoIrL2** or **IrL1** (0.1 μM) at 37℃ for 4 h and then co-incubated with DCFH-DA (10 μM, λ_{ex} = 488 nm, λ_{em} = 500-560 nm), MitoSOX Red (1 μM, λ_{ex} = 488 nm, λ_{em} = 550-610 nm) or JC-10 (5 μM, λ_{ex} = 488 nm, λ_{em} = 550-610 nm).

4T1 cells were seeded in the glass bottom cell culture dishes and cultured for 24 h. The medium was replaced with fresh one containing **MitoIrL2** or **IrL1** (0.1 μM). After incubation for 4 h, the cells were exposed to photoirradiation (white light, 6 mW/cm^2, 15 min). After treatment, the cells were washed twice with cold PBS, fixed with 4% paraformaldehyde for 15 minutes, then permeated with 0.1% Triton and blocked with 5% BSA for 30 minutes. Followed by CRT primary antibody incubation at 4℃ for 2 h, the cells were incubated by Alexa Fluor 488 secondary antibody for 1 h and incubated by Hoechst 33342 for another

10 minutes.

Cytotoxicity assay

Cells were seeded in a 96-well plate at a density of 1×10^4 cells per well and cultured for 24 h. Then cells were treated by complexes with different concentration. The cell viability assay was performed by 3-(4,5-dimethyl-2-thiazolyl)-2,5-diphenyl-2-H-tetrazolium bromide (MTT) method. The OD570 was accordingly measured by a microplate reader. The IC_{50} value was calculated finally.

The experiment is divided into the following three groups:

1. Dark. The cells were incubated with complexes for 24 h in the dark.
2. Normoxia + Light. The cells were incubated with complexes for 4 h in the dark and then exposed to photoirradiation (white light, 6 mW/cm^2, 15 min).
3. Hypoxia + Light. The cells were incubated with complexes for 3 h in the dark and then sealed in an anaerobic air bag 1 h before irradiation (white light, 6 mW/cm^2, 15 min) to create a hypoxic condition. Oxygen concentration was below 0.1% during irradiation.

Cell uptake

Cells were seeded in a 6-well plate at a density of 5×10^5 cells per well and cultured for 24 h. The medium was replaced with fresh one containing **MitoIrL2** or **IrL1** (10 μM). After incubation for 4 h, the cells were washed twice with cold PBS, trypsinized and centrifuged. The cell pellets were digested with HNO_3 (100 μL, 2 h), 30% H_2O_2 (50 μL, 1.5 h), and HCl (100 μL, 2 h) at 95 ℃, respectively. The solution was diluted with water, and the Ir content was determined by ICP-MS.

Flow cytometry analysis

RAW264.7 cells were seeded in 12-well plate and cultured for 24 h. The medium was replaced with fresh one containing **MitoIrL2** or **IrL1** (0.1 μM). After incubation for 4 h, the cells were washed twice with PBS and incubated with the culture medium containing ROS detection sensors for 20 min. The cells were washed twice with PBS and then exposed to photoirradiation (white light, 6 mW/cm^2, 15 min). Finally, the cells were collected and analyzed by flow cytometry (Angilent NovoCyte Quanteon flow cytometer).

RAW264.7 cells and BMDMs were seeded in 12-well plate and cultured for 24 h. The medium was replaced with fresh one containing LPS (100 ng/mL), IL-4 (20 ng/mL) and complexes (0.1 μM). After incubation for 4 h, the cells were exposed to photoirradiation (white light, 6 mW/cm^2, 15 min). After 24 h treatment, the cells were collected, washed twice with cold PBS and then incubated with different membrane surface antibodies for 30 min at 4℃. Finally, the cells were washed twice with cold PBS and analyzed by flow cytometry (Angilent NovoCyte Quanteon flow cytometer).

Western blot analysis

RAW264.7 cells were seeded in 6 cm plates and cultured for 24 h. The medium was replaced with fresh one containing LPS (100 ng/mL) and complexes (0.1 μM). After incubation for 4 h, the cells were exposed to photoirradiation (white light, 6 mW/cm^2, 15 min). After treatment, the cells were collected, washed twice with cold PBS and then lysed by cell lysis buffer with phosphatase and protease inhibitor for 30 min at 4℃. After measurement of protein concentration by Bradford assay, 40 μg proteins from different group were added into SDS-PAGE gel for electrophoresis and then transferred onto polyvinylidene fluoride (PVDF) membranes. After blocking by 5% BSA and followed by specific primary antibodies incubation at 4℃ overnight, the membranes were incubated by peroxidase-labeled HRP secondary antibodies for 1 h at

room temperature. The chemiluminescence HRP substrate peroxide solution was finally added and the signals were detected by the Gel Doc XR system (Bio-Rad).

Metabolic measurement

The cellular respiration rate was investigated using Seahorse Extracellular Flux analyzer (Agilent Technologies). RAW264.7 cells were seeded in 24-well plate and cultured for 16 h. The medium was replaced with fresh one containing LPS (100 ng/mL) and **MitoIrL2** (0.1 μM). After incubation for 4 h, the cells were exposed to photoirradiation (white light, 6 mW/cm^2, 15 min). Two hours later, the medium for OCR was replaced with XF DMEM base medium containing 2 mM glutamine, 1 mM pyruvate and 25 mM glucose, while the medium for ECAR was replaced with XF DMEM base medium containing 2 mM glutamine. The concentration of inhibitors: oligomycin (1 μM), FCCP (2 μM), rotenone (1 μM), antimycin A (1 μM), glucose (10 mM) and 2-DG (100 mM).

Detection of extracellular ATP and HMGB1

4T1 cells were seeded in 6-well plate and cultured for 24 h. The medium was replaced with fresh one containing **MitoIrL2** or **IrL1** (0.1 μM). After incubation for 4 h, the cells were exposed to photoirradiation (white light, 6 mW/cm^2, 15 min). After treatment, the supernatants were collected and then analyzed by different detection kit respectively.

Breast cancer mouse model

BALB/c and C57BL/6 female mice (4-6 weeks) of specific pathogen-free (SPF) grade were purchased from GemPharmatech (Nanjing, China) Co., Ltd., license No. SCXK (Jiangsu) 2018-0008. All the animal experiments followed the Guidelines for the Care and Use of Laboratory Animals, and were approved by the Institutional Animal Care and Use Committees of GemPharmatech, approval No. SYXK (Jiangsu) 2018-0027.

Each female BALB/c mouse subcutaneously was injected 1 × 10^6 4T1 cells. Mice were divided into three groups with different treatments as following (n = 6): PBS injection with irradiation (100 μL, white light, 100 mW/cm^2, 10 min); **MitoIrL2** injection with irradiation (4 mg/kg, 100 μL, white light, 100 mW/cm^2, 10 min); only **MitoIrL2** injection (4 mg/kg, 100 μL). Mice were intratumorally injected every two days. Mice weight and tumor volume were measured every two days. (Tumor volume = 0.5 × length × width^2)

Each female BALB/c mouse subcutaneously was injected 1 × 10^6 4T1 cells into the right hind limb area as primary tumor. Five days later, 1 × 10^6 4T1 cells were injected into the left hind limb area as distant tumor. Mice were divided into three groups with different treatments as following (n = 5): PBS injection with irradiation (100 μL, white light, 100 mW/cm^2, 5 min); **IrL1** injection with irradiation (4 mg/kg, 100 μL, white light, 100 mW/cm^2, 5 min); **MitoIrL2** injection with irradiation (4 mg/kg, 100 μL, white light, 100 mW/cm^2, 5 min). Mice were intratumorally injected every four days. Mice weight and tumor volume were measured every two days. (Tumor volume = 0.5 × length × width^2)

After the treatment, mice were sacrificed, and their major organs and tumors were performed histological analysis by means of hematoxylin-eosin (H&E) staining. Tumor tissue, spleen, lymph nodes and blood were also collected to assess immune response *in vivo*. Single-cell suspensions co-stained with antibody labeling were finally analyzed by flow cytometry (Angilent NovoCyte Quanteon flow cytometer).

Supporting figures

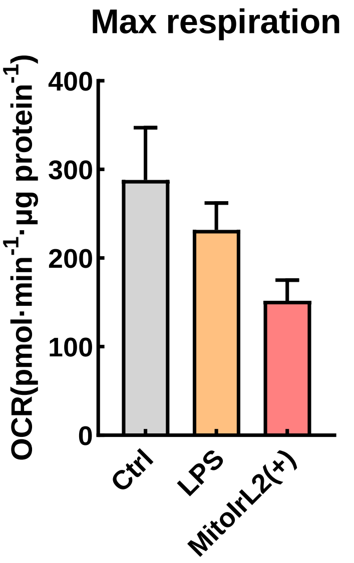

Figure S1. Quantification of maximal respiration from the kinetic profiles of OCR in RAW264.7 cells after treatment with LPS (100 ng/mL) or **MitoIrL2** (0.1 μM). (+): white light irradiation after 4 h incubation, 6 mW/cm^2, 15 min.

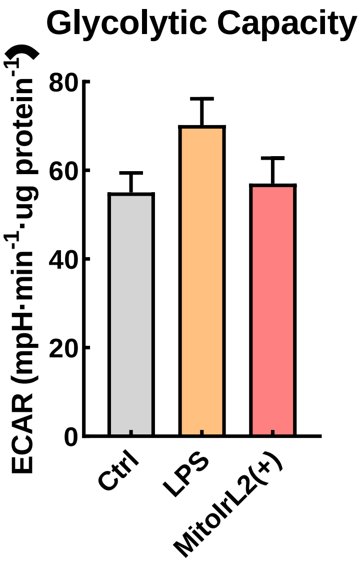

Figure S2. Quantification of glycolytic capacity from the kinetic profiles of ECAR in RAW264.7 cells after treatment with LPS (100 ng/mL) or **MitoIrL2** (0.1 μM). (+): white light irradiation after 4 h incubation, 6 mW/cm^2, 15 min.

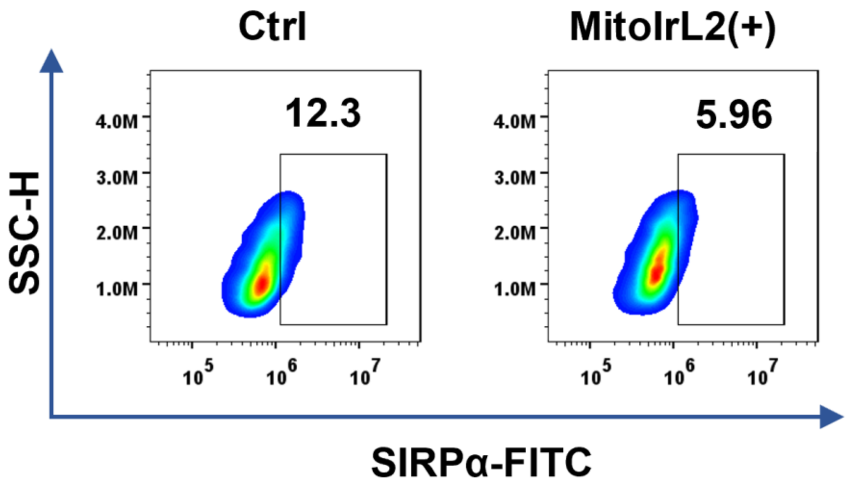

Figure S3. Flow cytometry analysis of SIRPα expression in BMDMs after the 24 h treatment with **MitoIrL2** (0.1 μM). (+): white light irradiation after 4 h incubation, 6 mW/cm^2, 15 min.

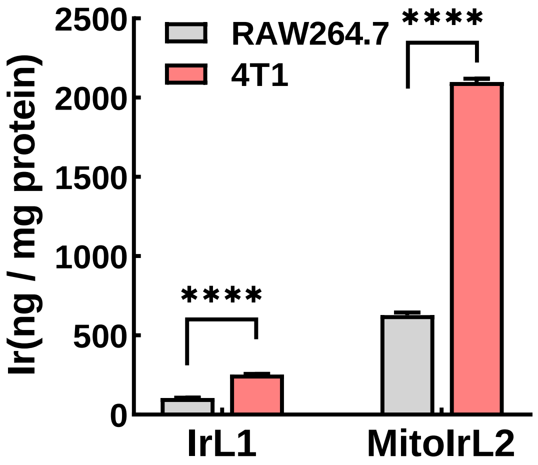

Figure S4. Iridium content in RAW264.7 and 4T1 cells determined by ICP-MS after 4 h incubation (10 μM).

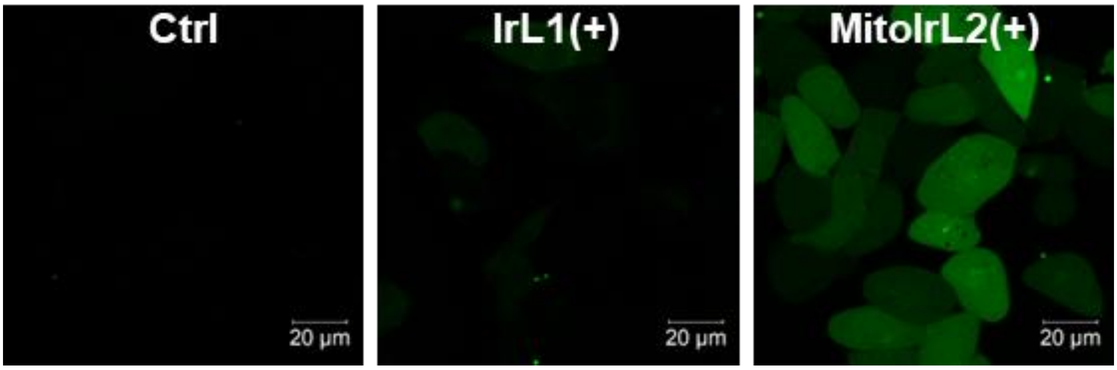

Figure S5. Confocal imaging of the ROS generation detected by DCFH-DA (λ_{ex} = 488 nm). Scale bar: 20 μm. (+): white light irradiation after 4 h incubation, 6 mW/cm^2, 15 min.

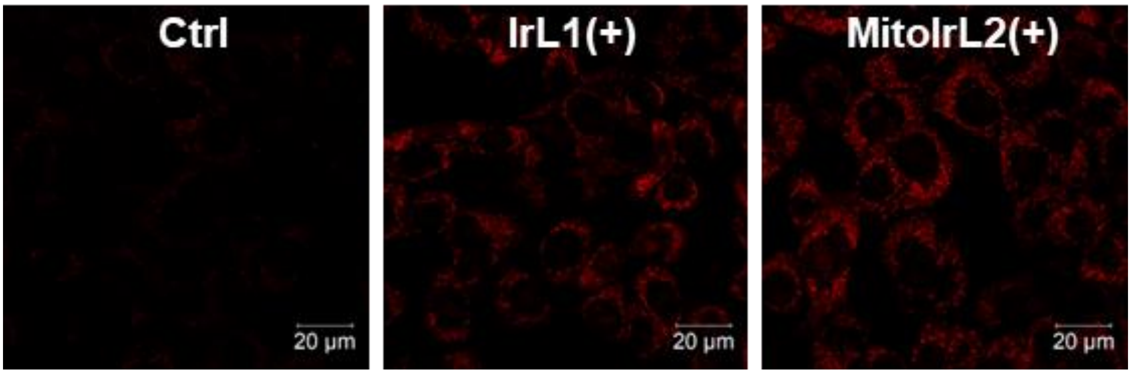

Figure S6. Confocal imaging of the MitoSOX generation detected by MitoSOX Red (λ_{ex} = 488 nm). Scale bar: 20 μm. (+): white light irradiation after 4 h incubation, 6 mW/cm^2, 15 min.

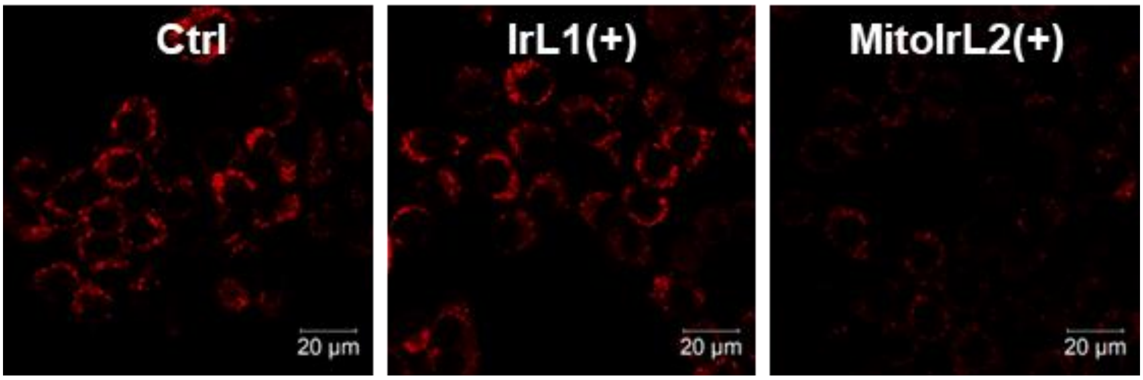

Figure S7. Confocal imaging of the JC-10 aggregates generation (λ_{ex} = 488 nm). Scale bar: 20 μm. (+): white light irradiation after 4 h incubation, 6 mW/cm^2, 15 min.

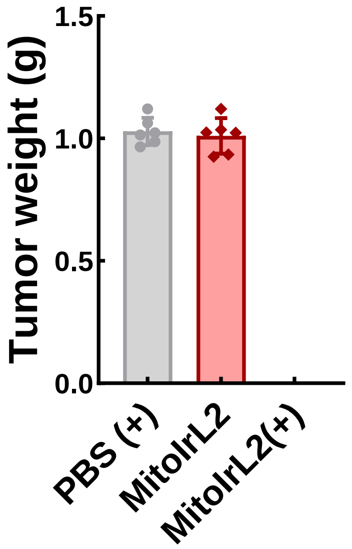

Figure S8. Weight histogram of tumors after different treatments (n = 6). (+): white light irradiation after 4 h incubation, 100 mW/cm^2, 10 min.

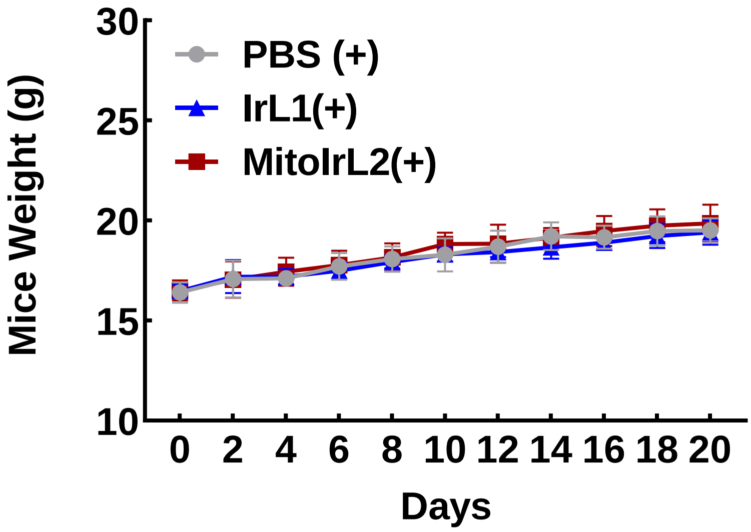

Figure S9. Weight curves of the mice with different treatments (n = 5). **IrL1**, **MitoIrL2**: 4 mg/kg. (+): white light irradiation after 4 h incubation, 100 mW/cm^2, 5 min.

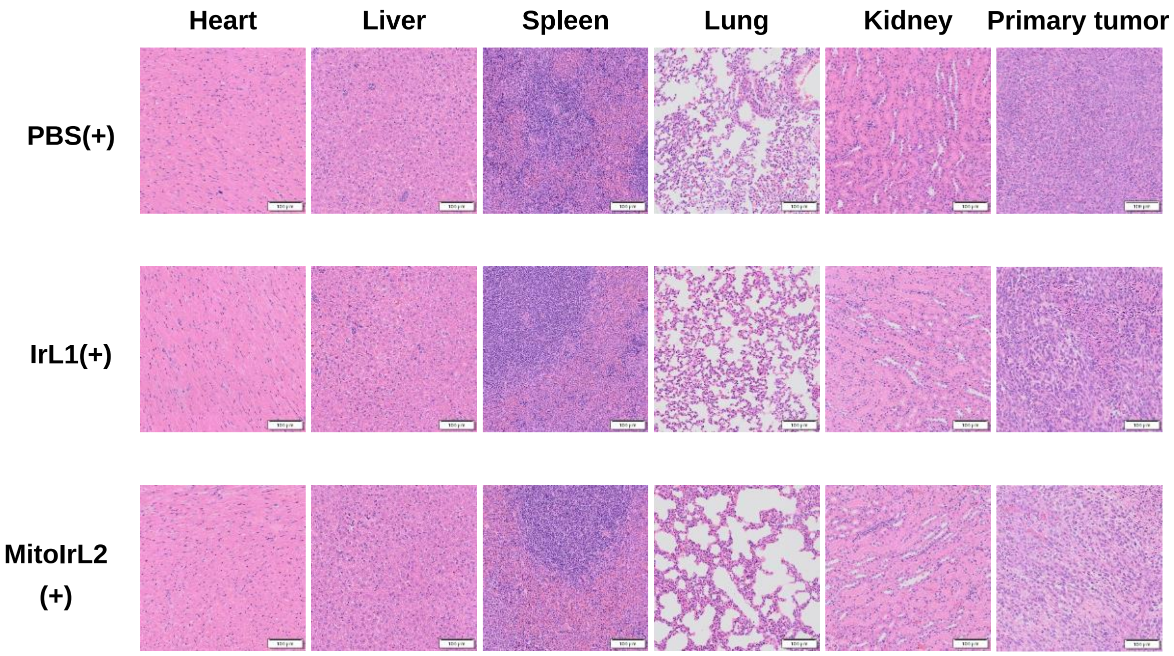

Figure S10. *In vivo* biosafety assay. H&E staining of major organs and primary tumors of mice from different treatment groups. Scale bar = 100 μm. **IrL1**, **MitoIrL2**: 4 mg/kg. (+): white light irradiation after 4 h incubation, 100 mW/cm^2, 5 min.

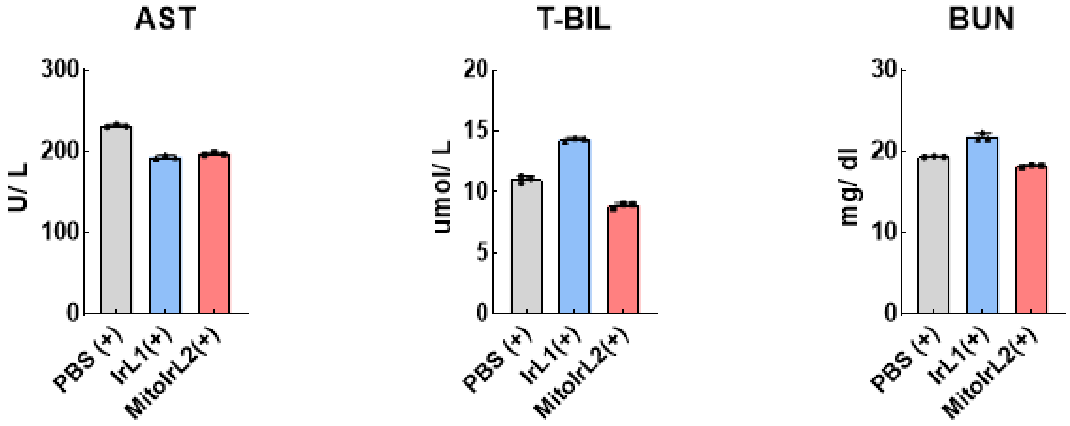

Figure S11. Blood biochemical analysis of the mice with different treatments (n = 3). **IrL1**, **MitoIrL2**: 4 mg/kg. (+): white light irradiation after 4 h incubation, 100 mW/cm^2, 5 min.

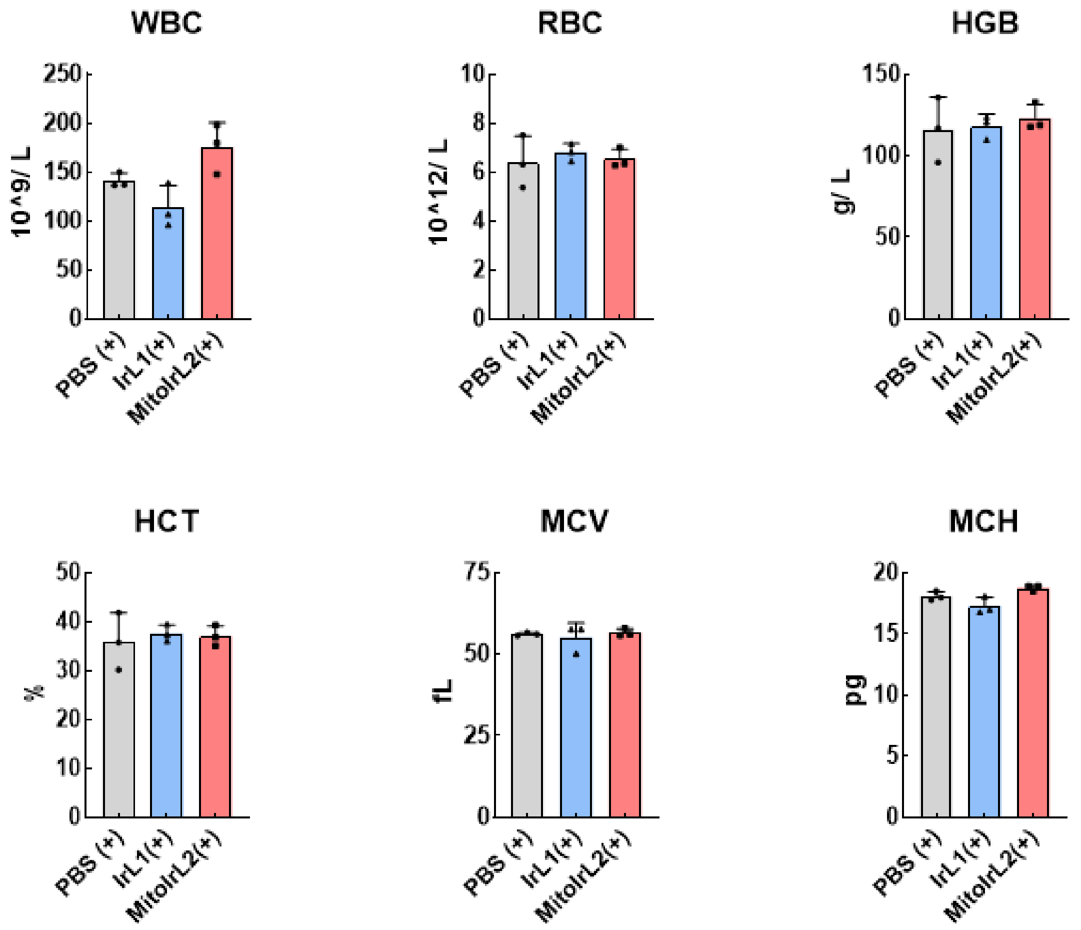

Figure S12. Hematological data of the mice with different treatments (n = 3). **IrL1**, **MitoIrL2**: 4 mg/kg. (+): white light irradiation after 4 h incubation, 100 mW/cm^2, 5 min.

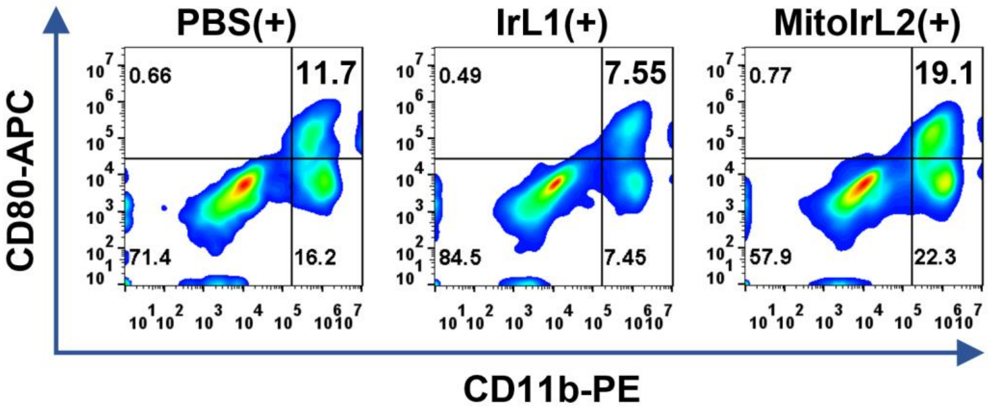

Figure S13. Flow cytometry analysis of M1 macrophages in primary tumor. **IrL1**, **MitoIrL2**: 4 mg/kg. (+): white light irradiation after 4 h incubation, 100 mW/cm^2, 5 min.

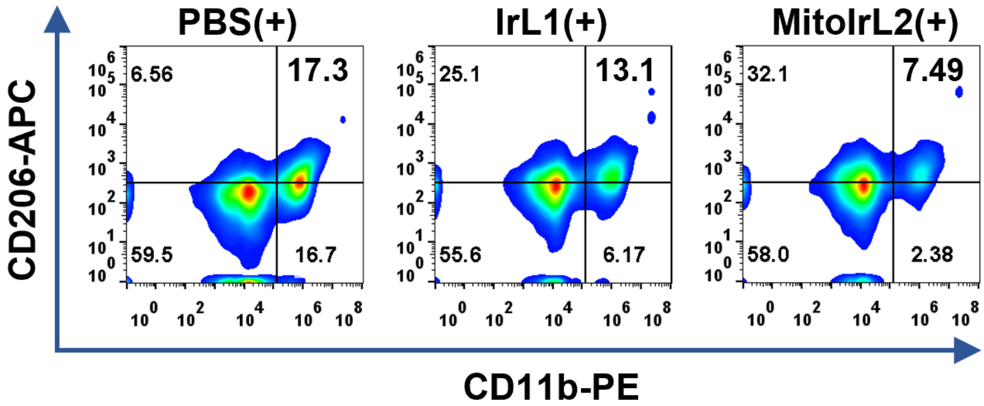

Figure S14. Flow cytometry analysis of M2 macrophages in primary tumor. **IrL1**, **MitoIrL2**: 4 mg/kg. (+): white light irradiation after 4 h incubation, 100 mW/cm^2, 5 min.

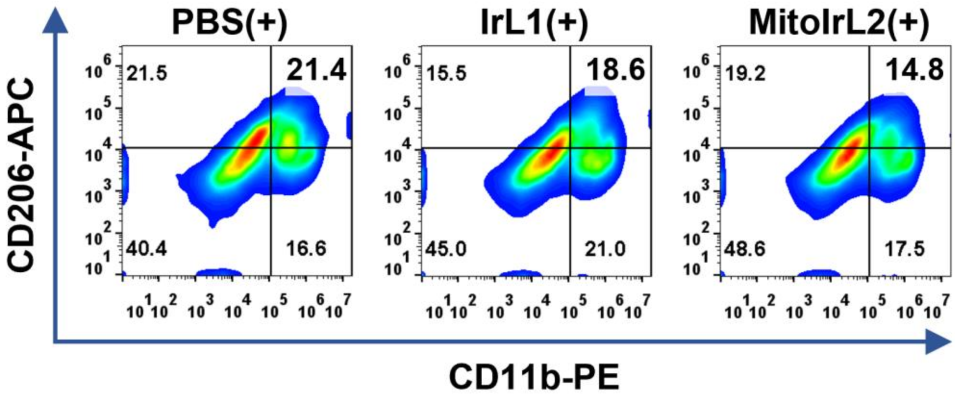

Figure S15. Flow cytometry analysis of M2 macrophages in distant tumor. **IrL1**, **MitoIrL2**: 4 mg/kg. (+): white light irradiation after 4 h incubation, 100 mW/cm^2, 5 min.

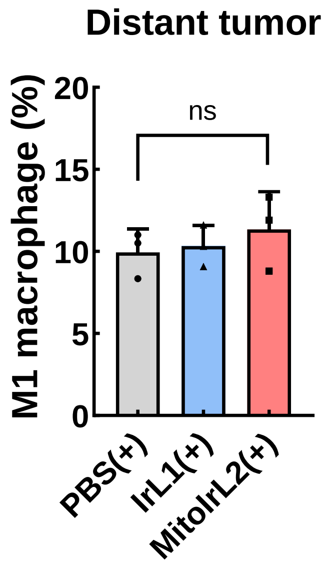

Figure S16. Flow cytometry analysis of M1 macrophages in distant tumor (n = 3). **IrL1**, **MitoIrL2**: 4 mg/kg. (+): white light irradiation after 4 h incubation, 100 mW/cm^2, 5 min.

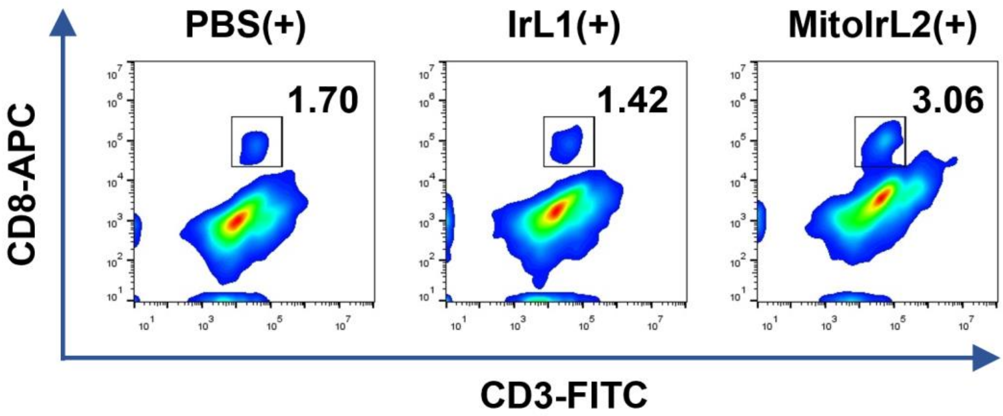

Figure S17. Flow cytometry analysis of CD3$^+$CD8$^+$ T cells in distant tumor. **IrL1**, **MitoIrL2**: 4 mg/kg. (+): white light irradiation after 4 h incubation, 100 mW/cm^2, 5 min.

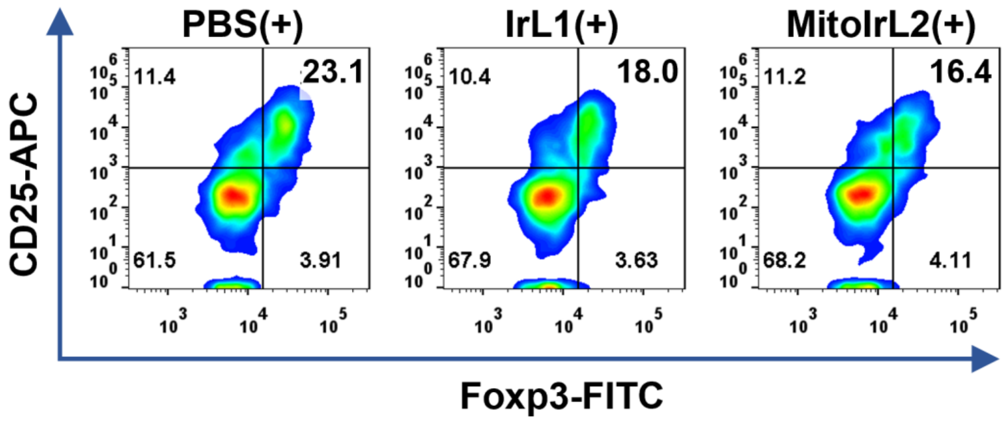

Figure S18. Flow cytometry analysis of Tregs in spleen. **IrL1**, **MitoIrL2**: 4 mg/kg. (+): white light irradiation after 4 h incubation, 100 mW/cm^2, 5 min.

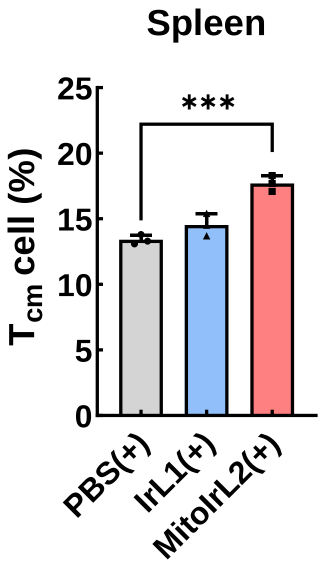

Figure S19. Flow cytometry analysis of central memory T cells in spleen (n = 3). **IrL1**, **MitoIrL2**: 4 mg/kg. (+): white light irradiation after 4 h incubation, 100 mW/cm^2, 5 min.

Supporting table

Table S1. IC_{50} values (μM) of **IrL1** and **MitoIrL2** against RAW264.7 and 4T1 cells.

Cell line	Complex	Dark[a]	Normoxia(+)[b]	PI[b]	Hypoxia(+)[c]	PI[c]
RAW264.7	**IrL1**	>16	0.56±0.22	>28	2.27±0.52	>7
	**MitoIrL2**	>16	0.33±0.05	>48	1.12±0.06	>14
4T1	**IrL1**	>16	0.23±0.03	>69	1.95±0.56	>8
	**MitoIrL2**	>16	0.11±0.02	>145	0.53±0.09	>30

[a] The cells treated with a 24 h incubation in darkness. [b] The cells treated with an incubation under normoxia (O_2 = 21%). [c] The cells treated with an incubation under hypoxia (O_2<0.1%). (+): white light irradiation after 4 h incubation, 6 mW/cm^2, 15 min. PI (phototoxicity index), the ratio of $(IC_{50})_{Dark}/(IC_{50})_{Light}$.
